# Ligands and Receptors Involved in the Sperm-Zona Pellucida Interactions in Mammals

**DOI:** 10.3390/cells10010133

**Published:** 2021-01-12

**Authors:** Lucie Tumova, Michal Zigo, Peter Sutovsky, Marketa Sedmikova, Pavla Postlerova

**Affiliations:** 1 Department of Veterinary Sciences, Faculty of Agrobiology, Food, and Natural Resources, Czech University of Life Sciences Prague, Kamycka 129, 165 00 Prague, Czech Republic; tumovalucie@af.czu.cz (L.T.); sedmikova@af.czu.cz (M.S.); 2 Division of Animal Sciences, University of Missouri, Columbia, MO 65211, USA; zigom@missouri.edu (M.Z.); sutovskyp@missouri.edu (P.S.); 3 Department of Obstetrics, Gynecology and Women’s Health, University of Missouri, Columbia, MO 65211, USA; 4 Laboratory of Reproductive Biology, Institute of Biotechnology of the Czech Academy of Sciences, BIOCEV, Prumyslova 595, 252 50 Vestec, Czech Republic

**Keywords:** spermatozoa, zona pellucida, gamete interaction, sperm-ZP receptors, ZP-ligands

## Abstract

Sperm-zona pellucida (ZP) interaction, involving the binding of sperm surface ligands to complementary carbohydrates of ZP, is the first direct gamete contact event crucial for subsequent gamete fusion and successful fertilization in mammals. It is a complex process mediated by the coordinated engagement of multiple ZP receptors forming high-molecular-weight (HMW) protein complexes at the acrosomal region of the sperm surface. The present article aims to review the current understanding of sperm-ZP binding in the four most studied mammalian models, i.e., murine, porcine, bovine, and human, and summarizes the candidate ZP receptors with established ZP affinity, including their origins and the mechanisms of ZP binding. Further, it compares and contrasts the ZP structure and carbohydrate composition in the aforementioned model organisms. The comprehensive understanding of sperm-ZP interaction mechanisms is critical for the diagnosis of infertility and thus becomes an integral part of assisted reproductive therapies/technologies.

## 1. Introduction

Mammalian fertilization is a species-specific event that involves a series of interactions between sperm protein molecules and zona pellucida (ZP) glycoproteins of the oocyte. The initial gamete interaction, also known as the primary binding of the spermatozoa to the ZP of the oocytes, is facilitated by the complementary sperm and zona surface molecules.

To gain the ability to bind to the ZP of an oocyte, spermatozoa undergo a sequence of post-testicular maturation events resulting in changes in the sperm protein composition, especially those localized to the sperm plasma membrane. Ejaculated spermatozoa have a fully differentiated morphology with a myriad of different protein molecules present on their surface [1,2,3]. During sperm transit through the female reproductive tract, the protein composition of the sperm plasma membrane changes dramatically, adapting spermatozoa to survival in the uterine environment [4] with the final step of capacitation leading to exposure of the receptors on the sperm surface responsible for ZP binding [5,6]. The sperm surface proteins are complementary to the oligosaccharide chains that decorate the ZP of the oocyte. Spermatozoa bind the ZP carbohydrate moieties via their membrane protein receptors resulting in, for most, part species-specific gamete recognition (reviewed by Clark [7]).

The differences in ZP carbohydrate moieties and sperm surface proteins are considered the main factor in the species specificity of sperm-ZP recognition and binding. While the concept of strict species-specificity applies to mice [8] and humans [9], this does not hold true for domestic animals such as pigs and cattle [10,11,12]. 

The initial interaction between the spermatozoa and oocyte takes place at the level of ZP. Therefore, receptors on the surface of capacitated spermatozoa are key to the fertilization process. The species-specificity of the sperm-ZP interaction can be ensured on the one hand by the presence of a certain receptor and, on the other hand, by a particular glycosylation pattern of the ZP. 

This review updates current knowledge about proteins and glycans involved in sperm-ZP interactions and proposed candidate receptors in thoroughly-investigated mammalian species, including mice, humans, porcine, and bovine. Determinants involved in the sperm-ZP binding regulate signal transduction resulting in subsequent acrosomal exocytosis (AE), sperm-ZP penetration, and gamete fusion during successful fertilization.

## 2. Zona Pellucida Glycoproteins

Zona pellucida (ZP) plays an important role in the oocyte lifespan providing mechanical protection [13] and defense against polyspermic fertilization by directly modulating sperm function [14,15]. The mammalian ZP is composed of three to four glycoproteins most commonly designated ZP1, ZP2, ZP3, and ZP4, with inter-species differences addressed below (Table 1). Four mammalian ZP glycoproteins are the products of three genes: *ZPA*, *ZPB*, and *ZPC* [16]. Phylogenic studies revealed that ZP2, encoded by *ZPA* and ZP3, coded *ZPC* is common in all the mammalian species so far investigated; meanwhile, ZP1 and ZP4 are products of the common progenitor *ZPB* gene, a duplication event that occurred during the evolution of the amniotes [17,18], see Table 1. Some authors differentiate *ZPB* paralogues into (*ZP1*/*ZPB1*) coding ZP1 and (*ZPB*/*ZPB2*) coding ZP4 [19]. In newer literature, genes encoding four ZP glycoproteins are termed *ZP1-4* to avoid nomenclature confusion [20], which is in accordance with HUGO nomenclature. From here on, we will use the HUGO nomenclature of ZP glycoproteins. Depending on species, either ZP1 or ZP4, or both are present. Synthesis of ZP glycoproteins was attributed to the growing oocyte in mice [13] whereas, in humans and other species (e.g., domestic pig, cattle, rabbit, and dog), granulosa/cumulus oophorus cells contribute to the synthesis and deposition of ZP as well [20]. ZP glycoproteins are conserved throughout the mammalian species sharing a high amino acid sequence identity between individual ZP1-4 homologs.

### 2.1. ZP Glycoproteins in the Mouse Model

In the best-studied animal model, a mouse, ZP is composed of three glycoproteins: mZP1 (200 kDa, dimer), mZP2 (120 kDa, monomer), and mZP3 (83 kDa, monomer) [23]. mZP1 shares the domain architecture with ZP4 that is expressed in other mammals such as human, pig, bovine, and dog (see relevant references in Fahrenkamp et al. [15]), and their genes are considered paralogous [22,24]. *ZP4* (*ZPB*/*ZPB1*) is a pseudogene in mice and therefore not expressed. The basic structural elements of murine ZP are repeating fibers formed by a pair of glycoproteins mZP2 and mZP3 (heterodimers) linked together by a dimer of mZP1 glycoprotein [23,25]. The estimated molar ratio of ZP1/ZP2/ZP3 is 1:4:4 [41]. Functional ZP glycoproteins consist of domains, including the signal peptide, ZP “domain” modules responsible for ZP polymerization, the consensus protease cleavage site, and a GPI-anchor [21]. ZP1 and ZP4, on top of the aforementioned domains, also contain the trefoil domain.

### 2.2. ZP Glycoproteins in the Humans

Contrary to the mouse, humans express all four ZP genes resulting in four ZP glycoproteins termed hZP1, hZP2, hZP3, and hZP4 [28]. hZP1 and hZP4 are paralogs, and their amino acids sequences share 47% identity. Human hZP1, hZP2, hZP3 amino acid sequences show 68%, 58%, and 68% homology with mouse mZP1, mZP2, and mZP3 glycoproteins, respectively (https://blast.ncbi.nlm.nih.gov/). Comparing the amino acid sequences between human ZP2, ZP3, and ZP4 and porcine glycoprotein homologs, there is 64%, 74%, and 68% sequence identity [27]. SDS-PAGE analysis revealed hZP2 as a 120 kDa band, hZP3 as a 58 kDa band, and the 65 kDa band contained both hZP4 and hZP1 [26]. The assembly of ZP glycoproteins into a matrix has been studied in a mouse model and was discussed above. It was reported recently that a frameshift mutation in the human *ZP1* gene caused primary female infertility as a result of the absence of the ZP2-ZP3 filament crosslinking and the inability to form a stable ZP matrix [29]. 

### 2.3. ZP Glycoproteins in the Pig Model

Porcine ZP is composed of three ZP glycoproteins, pZP2-4. *ZP1* is a pseudogene in the pig, and therefore ZP1 is not expressed. SDS-PAGE analysis revealed pZP2 (*ZPA*/PZPL) as a 90 kDa band that splits under reducing conditions into two smaller bands of 65 kDa and 25 kDa [31,32,33,36]. Both pZP3 (*ZPC*/ZP3-β) and pZP4 (*ZPB*/ZP3-α) migrated as 55 kDa protein bands [38]. pZP3 and pZP4 make about 80% of total porcine ZP glycoproteins [30,32]. The pZP2 and mouse mZP2 homologs share a 55% amino acid sequence identity, while pZP3 and mouse mZP3 share a 66% amino acid sequence identity (https://blast.ncbi.nlm.nih.gov/). The pZP4 was implied to have the same function as the mZP1 paralogue [35,37]. It was later predicted that similar to mice, pig ZP filaments are formed by pZP3 and pZP4 heterodimers, crosslinked with pZP2 based on their estimated molar ratio of 1:6:6 (pZP2:pZP3:pZP4) [34].

### 2.4. ZP Glycoproteins in the Bovine Model

Similarly, as in the pig, three glycoproteins were identified in bovine ZP, termed bZP2 (*ZPA*), bZP3 (*ZPC*), and bZP4 (*ZPB*) [39], and the *ZP1* is a pseudogene. Furthermore, SDS-PAGE analysis of deglycosylated ZP glycoproteins showed that bZP2 migrated at 76 kDa, bZP3 at 47 kDa, and bZP4 at 68 kDa. Similar to the domestic pig, bZP2, under reducing conditions, split into two smaller bands of 63 kDa and 21 kDa [39]. Amino acid sequences of bovine ZP glycoproteins show high similarity to their pig counterparts, i.e., 78%, 84%, and 76% for ZP2, ZP3, and ZP4, respectively (https://blast.ncbi.nlm.nih.gov/). bZP4 was found to have the strongest sperm-binding activity among the components, while bZP3 had about one-sixth that of bZP4 [40]. The estimated molar ratio of bZP2/bZP3/bZP4 in bovine is 1:2:1 [41].

## 3. Carbohydrate Structure and Glycosylation of ZP Glycoproteins

All ZP glycoproteins are highly heterogeneous due to post-translational modification by glycosylation of serine/threonine (O-linked glycosylation) and asparagine (N-linked glycosylation) residues, which are mostly sulfated and sialylated. Structures of the glycan portion of ZP proteins have been characterized by in-depth and reviewed in-detail [7,42,43,44]. The carbohydrate content of ZP is estimated at 15–54% *(w*/*w)*, and its heterogeneity is reflected as sets of trailing spots on 2-DE electrophoretograms. The glycosylation sites of individual oligosaccharides and cognate carbohydrate-binding proteins are involved in the sperm-ZP binding in many species in a species-specific manner [45,46,47].

In the 1990s, the sugar structures of ZP have deducted from lectin-binding studies. Some conserved carbohydrate structures were found in almost all species investigated, such as mannose and N-acetylglucosamine that are common components of the core of N-linked oligosaccharides [48,49,50]. On the other hand, β-galactose was found in mouse and bovine but not in porcine ZP [51]. Terminal N-acetylgalactosamine and α-galactose residues constitute minor components in murine and bovine ZP, whereas porcine N-glycans are lacking these N-acetylgalactosamine and α-galactose residues [45]. Human ZP also contains mannosyl, N-acetylglucosaminyl, and β-galactosyl residues and βGal-(1–3)GalNAc sugar sequences that are exposed only after removing terminal sialic acid residues [49]. Sialyl-Lewis^x^ structures are uniquely present in human ZP [52].

### 3.1. Glycosylation in the Mouse Model

The basic structure of N-linked oligosaccharides (complex-type) in mice is similar to porcine ZP [53,54]. Also, bovine N-linked glycans show practically the same structure as their murine and porcine homologs [55]. Species-specific differences are most obvious in the structure of neutral N-linked carbohydrates [56]. In the pig and cattle, neutral oligosaccharides represent about 25% of the total carbohydrate portion, whereas in the mouse they are present at less than 5%. Variations in other species are in di-, tri-, tetra-antennary chains, sulfation, and sialylation. The number of sulfated lactosamine repeats and degree of sialylation in both N- and O-glycans are the causes of enormous heterogeneity of the ZP glycoproteins in all species [45,57].

Mouse ZP contains N-linked oligosaccharides with high-mannose and complex-type structures (such as di-, tri-, and tetra-antennary branched N-glycans) as well as O-linked oligosaccharides [58]. The mZP oligosaccharides are complexes containing fucose residues [51] and form mainly acidic tri- and tetra-antennary chains containing lower amounts of sulfates and sialic acids in the N-linked chains [51,58,59]. N-glycans are fucosylated and elongated by non-branched N-acetyllactosamine chains. Acidic glycans contain sialic acids at the nonreducing end or sulfates in the C-6 position of the N-acetylglucosamine residues of the lactosamine repeats [45,55]. N-acetyl-D-lactosamine (LacNAc), sialized LacNAc, and terminal N-acetylglucosamine (GlcNAc) were found as terminal units of N-linked oligosaccharides. In O-linked oligosaccharides, the majority were core-2 type O-N-acetylgalactosamine [58], with mainly sialic acid found as a terminal unit [60]. Mouse ZP glycoproteins are composed of 16 potential N-glycosylation sites, with 15 of them being actually occupied [61]. The mZP1 contains four, mZP2 six and mZP3 six N-glycosylation sites. Mouse ZP has many additional potential O-glycosylation sites that are less utilized. There are as many as 82 potential O-linkage sites in mZP1, 84 in mZP2 and 58 in mZP3 [61]. mZP1 is more O-glycosylated than N-glycosylated, whereas mZP2 is predominantly N-glycosylated, with low or no O-glycosylation, and mZP3 is more N-glycosylated with relatively low O-glycosylation [61].

### 3.2. Glycosylation in the Humans

The glycan profile of human ZP is unique compared to other mammalian species [62]. Even though the lectin studies initially indicated a high content of D-mannose in human ZP [49], ultrasensitive mass spectrometric analyses revealed the absence of the high-mannose type chain [63]. Human N-linked ZP glycans have bi-, tri, and tetra- antennary fucosylated complex-type structures, and are terminated with sialyl-Lewis^x^ (SLEX) and sialyl-Lewis^x^-Lewis^x^. O-linked glycans in human ZP are core-1, and -2 type O-N-acetylgalactosamine, but only core-2 type possess terminal SLEX [63]. Sialyl-Lewis^x^ sequences on O- and N-glycans are important for sperm-oocyte binding. Human sperm-egg binding depends primarily on the recognition of terminal SLEX that is expressed on about 85% of all N-glycans [52,63]. SLEX was found to be expressed more densely in the outer region of ZP than in the inner layer [52]. In human hZP2, hZP3 and hZP4 glycoproteins, the N-linked glycosylation is predominant. Although N-linked glycosylation occupies 37%, 27% and 18% of the molecular mass of hZP2, hZP3, and hZP4, respectively, the percentages of O-linked glycosylation are only 8% for hZP2, 9% for hZP3 and hZP4 seems to be without O-linked glycosylation [26].

### 3.3. Glycosylation in the Pig Model

As in the other species previously discussed, porcine ZP glycoproteins are highly heterogeneous due to varied amounts of sialylated and/or sulfated poly-N-acetyllactosamine [64]. N-linked chains are composed of neutral and acidic chains at a molar ratio of about 1:3 that constitute di-, tri- and tetra-antennary N-glycans complex with α-fucosyl residue in the innermost N-acetylglucosamine [65]. The main neutral N-glycans of porcine ZP glycoproteins belong to the di-antennary fucosylated glycans containing N-acetyllactosamine chains [45] and are implicated in sperm-oocyte recognition [34]. Highly sulfated acidic N-glycans consist of poly-N-acetyllactosamine sequences of different lengths, sulfated at the C-6 position of GlcNAc [54]. In contrast to the N-glycans of ZP in cyclic sows, a lower degree of glycan sulfation in the prepuberal zona pellucida has been reported [66]. N-linked glycans contain fucose residues but no high mannose chains [51]. The largest ZP glycoprotein in the pig, pZP2 has six, pZP3 three, and pZP4 five potential N-glycosylation sites. In addition, pZP4 contains three and pZP3 six potential O-glycosylation sites [37]. Sugar-mapping of pZP4 glycopeptides has revealed that all three potential N-glycosylation sites Asn203, Asn220, and Asn333 of the mature pZP4 carry neutral bi-antennary N-glycans, whereas only Asn220 is also glycosylated with neutral tri- and tetra-antennary chains. At least one disulfide bond between the neighboring cysteine residues Cys224 and Cys243 has been localized in the N-terminal part of pZP4 [45,57]. O-linked glycans comprise 9 neutral and 26 acidic unbranched chains of core-1 O-N-acetylgalactosamine type [67]. Similar to N-linked glycans, the O-linked glycans are sulfated at the C-6 position of GlcNAc and/or sialylated. The N-glycosylation of porcine ZP glycoproteins, which occurs during meiotic maturation is crucial in sperm-ZP interactions, including sperm binding to ZP and induction of AE in ZP-bound sperm [68]. Nevertheless, the binding and induction of AE in boar spermatozoa do not require the participation of terminal Galα1-3Gal sequences [69].

### 3.4. Glycosylation in the Bovine Model

Thus far, only N-linked glycans have been reported in bovine ZP [51]. Bovine ZP glycoproteins are contained with 23% of neutral carbohydrate chains, of which the main constituent is high-mannose-type oligosaccharide structure, and 77% of acidic chains with a high content of sialic acid as opposed to the high content of sulfation that is typical for the pig [59]. Bovine ZP glycans are therefore more similar to those of the mouse than the pig and human. The acidic N-linked glycans of bovine ZP contain di-, tri- and tetra-antennary sialylated complex-type structures with a fucose residue at their reducing ends [51]. Molecular cloning of bovine ZP revealed five potential N-glycosylation sites in bZP4 (*ZPB)*, three potential glycosylation sites in bZP3 (*ZPC*), and four potential N-glycosylation sites in bZP2 (*ZPA*) [40,70]. Further studies confirmed bZP2 being N-glycosylated at Asn83, Asn191, and Asn527 [71], and bZP2 being N-glycosylated at Asn124, and Asn146 [70].

## 4. Sperm-Zona Pellucida Interaction Ligands

It has been generally accepted that the interaction between the spermatozoa and the oocyte ZP during fertilization is a multi-step process, including the initial sperm attachment to the ZP surface glycoproteins, also known as the primary sperm-ZP binding, resulting in the induction of AE, reinforced binding to ZP also known as the secondary sperm-ZP binding, sperm penetration through the ZP, and the adhesion and fusion of the sperm plasma membrane with the oolemma [72,73,74,75]. The primary sperm-ZP binding event is mediated by complementary protein molecules (receptors) on the sperm surface, which interact with lectin-like proteins and/or carbohydrates/glycoconjugates of ZP [7]. A number of the candidate sperm receptors that are discussed in the following section, possess a lectin-type affinity for specific sugar residues of ZP. The sperm interactions with the ZP glycoproteins are species-specific, mainly due to the differences in ZP glycosylation (see the previous section). As will be discussed in the following section, sperm molecules involved in the primary sperm-ZP binding originate from both spermatogenic cells and from seminal plasma produced by accessory sex glands; they localize to the apical region of the anterior part of the sperm head acrosome. On the contrary, molecules involved in the secondary binding originate predominantly from spermatogenic cells and localize mainly to the inner acrosomal membrane which is exposed by acrosomal exocytosis after primary sperm-ZP binding [76].

The last two decades, however, showed that this simplistic model might not reflect the complexity of this fertilization step in its entirety. In the late 1980s’, Fraser at el. [77] noted a higher incidence of acrosomal loss in the capacitation promoting media, which was later elaborated by Kim and Gerton [78] to conclude that AE is a continuously variable process initiated under capacitating conditions, and once spermatozoa encounter the ZP, the rate of AE is accelerated. Therefore, the idea arose that ZP might not be the only physiological inducer of AE, and rather than ZP triggering AE, it accelerates the progress of AE. On the side of spermatozoa, the concept got even more perplexing when it was reported that some acrosomal matrix proteins with ZP-binding affinity such as ZAN, ACR, ACRBP, ZPBP1, and ZP3R traffic to the sperm head surface during sperm capacitation and thus might participate in the initial (primary) sperm-ZP binding as well [79,80,81]. It is thus plausible that sperm capacitation primes spermatozoa for AE, and sperm-ZP adhesion induces it.

### 4.1. ZP Ligands for Sperm Binding in the Mouse Model

The mouse has been the most extensively studied animal model for sperm-ZP interactions since the 80s. It was shown early that epididymal, acrosome intact spermatozoa were binding mZP3 resulting in subsequent induction of AE [82,83,84]. At that time, it was believed that α-Gal residues at the nonreducing end of the O-linked chains within the C-terminus of mZP3 were being recognized by acrosome intact spermatozoa [13,85,86], pinpointed to the region Ser329 to Ser334 of mZP3 [87]. This model was, however, not supported by the results of Thall et al. [88], where galactosyltransferase-KO female mice lacking α-Gal residues remained fertile. Instead, β1-4 linked Gal residues of LacNAc sequence, with or without α1–3 Gal cap, were thought to be responsible for approximately 80% of murine sperm-ZP binding [89,90,91]. On the other hand, AE spermatozoa were preferentially binding mZP2 [92], which was later confirmed, and a sequence of about 100 amino acids near the N-terminus was shown to be involved in this interaction [93]. The idea that spermatozoa are intact when they encounter ZP arose from the studies of Saling et al. and Saling and Storey [94,95] and had become a widely accepted, long-lasting paradigm of mZP3 serving as the primary ZP-sperm ligand for acrosome intact spermatozoa that can induce AE while mZP2 served as the secondary sperm ligand. This was mainly because epididymal, as opposed to ejaculated spermatozoa, are still widely used in the mouse model, which does not completely reflect the situation in vivo because of the lack of epididymal sperm exposure to seminal plasma. This concept was often challenged, and as previously noted, Kim and Gerton [78] proposed that by the time capacitated spermatozoa reached ZP, they were already committed to AE. Baibakov et al. [96] reported that the mere binding of acrosome intact spermatozoa to ZP is not sufficient for the induction of AE and proposed a different model of AE. Other authors reported that AE starts as soon as spermatozoa reach cumulus cells [97], and this concept was finally refuted with the study of Inoue et al. [98], where the authors reported that spermatozoon extracted from perivitelline space could fertilize another zona-enclosed oocyte. Due to these new findings, the place of AE induction, inducers of AE, as well as the mechanism by which the acrosome mediates sperm-oocyte interaction, still remains to be resolved [99,100,101]. As noted previously, the nature of initial sperm-ZP interactions relies primarily on the recognition of carbohydrate moieties present on the ZP by lectin-like binding receptors on the sperm head (carbohydrate-dependent model) [7]. Alternative molecular models for murine sperm-ZP binding were proposed including, protein-protein interactions (carbohydrate-independent) model and the redundant, perhaps synergistic carbohydrate-protein and protein-protein interactions (domain-specific) model [102,103].

### 4.2. ZP Ligands for Sperm Binding in the Human

Human gametes have recently become a predominant study subject for the investigation of sperm-ZP interactions. The role of human ZP glycoproteins in sperm binding and induction of AE was exhaustively reviewed in Gupta [20]. Studies using either native or *E. coli* or baculovirus-expressed recombinant hZP glycoproteins showed that more than one ZP glycoprotein is responsible for the binding of spermatozoa to the oocyte with the ability to induce AE. In fact, hZP1, hZP3 and hZP4 were all found to bind capacitated spermatozoa and to induce AE. hPZ3 and hZP4 seem to have distinct binding sites on capacitated spermatozoa [104]. N-linked glycans of hZP1, hZP3, and hZP4 were not found to be necessary for sperm-ZP binding; however, they are indispensable for the induction of AE [20]. As much as 79% of human sperm-ZP binding may rely on lectin-like interactions [105], predominantly mediated by the terminal carbohydrate sequence termed sialyl-Lewis^x^ (NeuAcα2-3Galβ1-4(Fucα1-3)GlcNAc) that is expressed on about 85% of all N-glycans [63]. Similar to the mouse model, hZP2 binds only to post-AE spermatozoa and is thought to serve as the secondary binding ligand [26,104,106]. Human ZP is believed to be the primary physiological inducer of AE in the oocyte-bound spermatozoa; however, this does not mean that it is the sole AE inducer [101].

### 4.3. ZP Ligands for Sperm Binding in the Pig Model

In the porcine model, pZP4 at its N-terminal region (Asp137 to Lys247) has been identified as the sperm-binding active fragment [65], and the pZP3/pZP4 heterocomplex is essential for the sperm-binding activity of glycoproteins [107]. The N-linked glycosylation at Asn203 and Asn230 of pZP4 was found to be vital for sperm-ZP binding [108], and the nonreducing LacNAc (Galβ1-4GlcNAc) residues of the tri- and tetra- antennary complex-type N-linked chains mediate the binding [12,64,109]. Interestingly, the sperm binding specificity changed to α-Man after AE [12]. The O-linked glycans on pZP3/pZP4 were also suggested to participate in sperm-ZP binding [37]. Since the β1-4 linked Gal residues of LacNAc sequence were found to be responsible for murine sperm-ZP binding as well, it is not surprising that murine spermatozoa can bind porcine ZP [69]. Of interest, porcine ZP appears to share certain surface glycans with rabbit erythrocytes, which may explain the ability of rabbit erythrocytes to bind both murine and porcine spermatozoa in a hybrid cell culture system, although unlike porcine spermatozoa, the mouse ones do not initiate AE upon such interaction [110]. The pZP3/pZP4 glycans are vital for the induction of AE [111], but since porcine spermatozoa may already initiate AE at contact with cumulus oophorus, ZP might not be the sole physiological AE inducer in this species [112].

### 4.4. ZP Ligands for Sperm Binding in the Bovine model

Similar to domestic pigs, the bZP3/bZP4 heterodimer mediates interactions with spermatozoa in bovine species [70,113], and native bZP4 has the highest sperm-binding activity among all of bZPs [40]. Nonreducing terminal α-mannosyl residues of the N-linked high-mannose-type chains play a vital role in bovine sperm-ZP binding [108,114], and the sperm-binding specificity does not change after AE, unlike in the pig [12]. N-glycosylation on Asn146 of bZP3 was found to be essential for bovine sperm-ZP binding [70]. The involvement of sialic acid in the sequence Neu5Ac(α2-3)Gal(β1-4)GlcNAc has also been implicated in bovine sperm-ZP binding [115]. Even though bZP was found to induce sperm AE in vitro [116], in vivo studies indirectly suggest that bZP might not be the only physiological inducer of AE [117,118].

## 5. Sperm Surface Receptors with ZP-Binding Affinity

**Figure 1:**
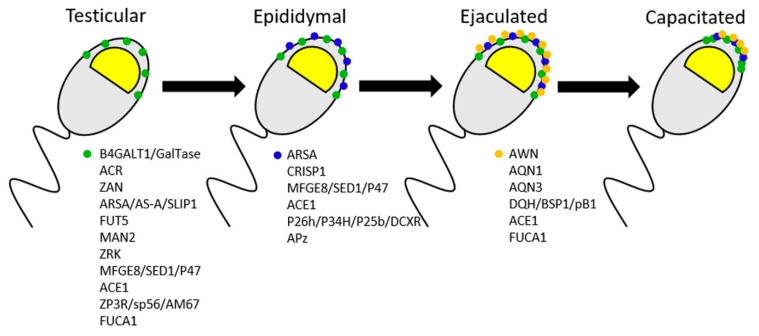
The incorporation of proteins with sperm-zona pellucida (ZP) binding affinity in the sperm surface during the sperm transit through the male reproductive tract. Green dots, blue dots and gold dots represent proteins with testicular, epididymal fluid, and seminal plasma origins, respectively. Proteins of testicular origin are incorporated in spermatozoa during spermatogenesis, while proteins originated in epididymal fluid, and seminal plasma are transferred to the sperm surface during the passage through the epididymis (epididymal maturation) and ejaculation, respectively. During sperm capacitation, redistribution of sperm-binding receptors occurs, guided by the formation of sperm membrane rafts. (B4GALT1/GalTase = galactosyltransferase; ACR = proacrosin/acrosin; ZAN = zonadhesin; ARSA/AS-A/SLIP1 = arylsulphatase A, sulfolipid immobilizing protein; FUT5 = α-1-3 fucosyltransferase; MAN2 = α-D-mannosidase; ZRK = zona receptor kinase; ACE1 = angiotensin-converting enzyme 1; FUCA1 = alpha-L-fucosidase; CRISP1 = cysteine-rich secretory protein; APz = adhesion protein z; AWN, AQN1, AQN3 = spermadhesins).

Sperm surface molecules with ZP-binding affinity have been studied for four decades. Additionally, many surface molecules have been proposed to serve as receptors for the primary sperm-ZP binding. The insertion of ZP-binding proteins into sperm plasma membrane occurs during spermatogenesis, followed by their translocation to the sperm surface during the epididymal maturation and addition of seminal plasma proteins at ejaculation (see Figure 1; relevant sperm proteins are detailed in the following sections and in Table 2). As mentioned previously, several known intra-acrosomal proteins with ZP-binding affinity translocate to the sperm surface during sperm capacitation, after which they can participate in the primary ZP binding. Sperm receptors involved in this binding are localized on the plasma membrane of the apical region of the capacitated sperm head. Similar to ZP glycoproteins, many of these sperm surface proteins are species-specific (see below). The known molecules with ZP-binding affinity reported in the mouse, humans, the pig, and the bovine, including their origin, localization, and binding specificity are summarized in Table 2.

### 5.1. Evolutionarily Conserved Mammalian Sperm-ZP Receptors and Other ZP-Binding Proteins 

First, we will discuss the ZP-binding molecules that are shared in the species reviewed. 

#### 5.1.1. Galactosyltransferase (B4GALT1/GalTase)

One of the first investigated and reported sperm-ZP binding receptors is a β1,4-Galactosyltransferase (B4GALT1/GalTase) has been implicated in sperm-ZP binding protein localized in the acrosomal cap in the mouse [119,255,256,257], pig [126,127], and also in bull [126,128,258]. B4GALT1 belongs to the glycosyltransferase enzyme family that catalyzes the transfer of glycosyl residue to the terminal sugar of a saccharide chain. Sperm B4GALT1 is a transmembrane protein that is incorporated into the plasma membrane during sperm development in the testis. Mouse sperm B4GALT1 binds galactose and N-acetylgalactosamine residues on terminal N-acetylglucosamine oligosaccharides of ZP3 glycoprotein [119]. By aggregation of B4GALT1, ZP3 induces subsequent acrosomal exocytosis of mouse and boar spermatozoa [93,119,126]. However, the presence of B4GALT1 is not essential for successful fertilization in the mouse, as demonstrated by the gene KO experiment in which the B4GALT1-null males were fertile. However, spermatozoa from B4GALT1-null males have a reduced ability to initiate AE as a response to ZP3 binding, but still retain the capability to bind to the coat of oocyte and fertilize it [259,260]. Although Tulsiani et al. [172] initially did not detect any B4GALT1 activity in the human sperm plasma membrane, a later study by Huszar et al. [125] found the B4GALT1 activity on the surface of human spermatozoa. Nevertheless, the precise localization of B4GALT1 on human spermatozoa has not yet been described.

#### 5.1.2. Proacrosin/Acrosin (ACR)

Another conserved ZP-binding sperm protein is proacrosin/acrosin (ACR). A fucose-binding protein has first been detected in the porcine spermatozoa by employing a specifically developed modified enzyme-linked-lectin-assay [261], and the N-terminal sequence of this fucose-binding protein identified it as ACR [262]. ACR is synthesized in its zymogen form, proacrosin, and is converted to its active form during capacitation via several intermediate forms [145,146]. ACR shows a high affinity to sulfate groups within the lactosamine repeats of N- and O- glycans of the ZP [45,262]. Although ACR has been described as a secondary binding receptor to ZP, abundant in the acrosomal matrix, its presence on the surface of human and boar sperm acrosomes [137,149,254] suggests that acrosin could also participate in primary sperm-ZP binding. Tanphaichitr et al. [80] showed that a portion of ACR is indeed transported to the sperm surface during capacitation. Proacrosin/acrosin has been reported in the acrosome of mouse spermatozoa as well [129,132]. Studies of ACR knock-out mice and rats showed that these animals were fertile despite a delay in the dispersion of the cumulus cells by ACR-null spermatozoa in both species [263,264] and delayed fertilization in the mouse [265]. The contribution of ACR to fertilization, however, may be more profound in other species. Dudkiewicz [266] reported that the fertilization rate was decreased in rabbits inseminated with spermatozoa pre-treated with anti-acrosin antibodies. In humans, the inhibition of acrosin by soybean trypsin inhibitor prevented spermatozoa from penetration of ZP in vitro [267]. Most importantly, contrary to rat and mouse ACR-KO ablation models, *ACR* gene ablation rendered male hamsters completely infertile due to a failure of sperm-zona penetration [268]. It appears that the mouse is rather an exception as the sperm acrosin activity is weaker when compared to other mammalian (rodent) species [269], suggesting it may not rely solely on acrosin. Furthermore, murine ZP of ~6.2 µm [270] is thinner when compared to other species, e.g., ~18 µm in the rabbit: [271,272], ~20 µm in the golden hamster [273], ~18 µm in pigs [11], ~16 µm in cattle [274] and ~16 µm in humans [275]. Limited information is available about the proacrosin/acrosin system in bull spermatozoa. Nevertheless, its presence in the acrosomal region of bull spermatozoa has been associated with sperm penetration through ZP [152].

#### 5.1.3. Zonadhesin (ZAN)

Another sperm surface protein with ZP-binding ability, zonadhesin (ZAN), is a multiple-domain protein [157,276,277] originally isolated from boar spermatozoa [157,158,159,278], and later reported in mouse [153], bull [159] as well as in human spermatozoa [155,156]. The ZAN is a transmembrane protein that is expressed during spermatogenesis in early spermatids [153,158] and is very quickly post-translationally modified by proteolytic enzymes [153,277]. The structure of ZAN shows significant amino acid sequence variations among mammalian species [277]. ZAN displays a multifunctional mosaic structure with domains such as an extracellular MAM domain, a mucin-like domain present in pathogens, a von Willebrand D-domain common in extracellular glycoproteins, and a domain homologous to epidermal growth factor (EGF). These domains are involved in multiple protein-protein cell interactions, including sperm-ZP binding [279]. ZAN also facilitates cell interactions in the male reproductive tract, for example, during spermatogenesis (between germline, Sertoli, and epithelial cells) or may act as a barrier to prevent nonspecific interactions between spermatozoa and other cells in the female reproductive tract, for instance, sperm adhesion in the oviduct [153].

#### 5.1.4. Arylsulphatase A (ARSA/AS-A)

Arylsulphatase A (ARSA/AS-A), also known as sulfolipid immobilizing protein (SLIP1) or p68, was reported in mouse, human, boar and bull spermatozoa [166,167,280,281,282,283]; however, the ZP-binding affinity in bovine is assumed based on other models. In the male reproductive system, ARSA is reported in three forms: (i) the intra-acrosomal form emerging at high levels during the formation of this organelle in spermatids, therefore of testicular origin, (ii) the surface-associated form that is expressed in the epididymal tissue and incorporated to the sperm surface during the epididymal passage, and iii) a free, secreted form in the epididymal fluid [160,161,162,163,166,283]. ARSA is an enzyme desulfating sulfoglycolipids, specifically targeting sperm sulfogalactosylglycerolipid (SGG) [284] during and after ejaculation [285]. The ARSA found on the sperm surface overlying the acrosome contains positively charged amino acids that promote binding to SGG, which is present in the mammalian testes and spermatozoa and implicated in sperm-ZP binding [281]. ARSA and SGG may co-interact with ZP3 via binding to sulfated sugar residues present on the oocyte ZP glycans [162,166,283]. Furthermore, the role of ARSA in sperm-ZP binding was shown by anti-ARSA IgG, which decreased mouse sperm-ZP binding in a dose-dependent manner [162,286].

#### 5.1.5. MFGE8/SED1/p47/Lactadherin

Mouse MFGE8/SED1 (a homolog to boar p47/lactadherin) is localized to the Golgi complex of spermatids, from which it is probably secreted. However, the predominant source of MFGE8 appears to be the initial segment of the caput epididymis where it is secreted by epithelial cells and coats the sperm head overlying the acrosome via intercalation of its discoidin/C domains into the sperm plasma membrane [189,190]. Mouse MFGE8 is a peripheral membrane protein homologous to a group of secreted proteins containing N-terminal Notch-like type II EGF (epidermal growth factor) repeats and C-terminal discoidin/F5/8 type C domains. These domains are responsible for MFGE8 attachment to the sperm membrane and the interaction with ZP [189,190]. The homolog of murine MFGE8 has also been reported in the pig, and, similarly, as in mouse, it behaves as a peripheral membrane protein [192]. Porcine MFGE8, previously referred to as p47 or lactadherin, was isolated from boar spermatozoa by affinity chromatography on immobilized ZP glycoproteins and homology to the short isoform of MFGE8 was determined [192,193]. Porcine MFGE8 was detected in the acrosomal region of testicular, epididymal, and in vitro capacitated spermatozoa [149,192]. The localization and expression of porcine MFGE8 change during post-testicular sperm maturation and capacitation [80,193]. The expression of porcine MFGE8 increases during the sperm transit from caput to cauda epididymis. MFGE8 was also reported as a minor constituent of adult boar seminal plasma [287], and therefore more MFGE8 may bind to the sperm surface during ejaculation. This stepwise MFGE8 acquisition is probably caused by the progressive accumulation of MFGE8 on the sperm surface [193]. Interestingly, porcine MFGE8 is also implicated in the binding to oviductal glycans that promote a sperm reservoir formation via their interaction with sulfated Lewis-X structures [288]. During capacitation, porcine MFGE8 appears to be unmasked by the release of coating proteins, possibly with a portion of MFGE8, resulting in the spreading from the apical ridge over the entire acrosomal region during sperm capacitation [193,289]. Of interest, MFGE8 was found to copurify with 26S proteasome [290], one of the proposed zona lysins [291], and a component of high-molecular -weight zona-binding complexes that will be discussed below. Furthermore, the capacitation related release of the sperm coating proteins as well as the relocation of MFGE8 from the apical ridge to the entire acrosome is modulated by 26S proteasome [289,292]. As mentioned earlier, porcine MFGE8 also has a mosaic structure organized into two N-terminal EGF-like domains followed by two tandem repeats with similarity to C1 and C2 domains found in blood clotting factors V and VIII, known to be involved in lipid binding. The second, the EGF-like domain, contains an integrin-binding sequence for cell adhesion [192]. MFGE8 was also found to be expressed on the acrosomal surface of intact human spermatozoa [191].

#### 5.1.6. ZP3R (Syn. sp56/AM67)

ZP3 binding protein ZP3R (syn. sp56/AM67) was first identified in mouse spermatozoa and initially localized to the acrosomal surface [207,208]; for reviews, see [79,293]. Intra-acrosomal localization of ZP3R was reported later [209,294]. Further study of ZP3R discovered that, during sperm capacitation, this protein translocated from the acrosomal matrix to the sperm plasma membrane [78,295]. ZP3R is expressed in testis during early spermiogenesis, and its N-linked carbohydrate side chains are trimmed during the differentiation to spermatids [209]. Even though unfertilized oocytes treated with recombinant ZP3R showed diminished binding of spermatozoa to the ZP [296], the *ZP3R*^−/−^ mice were reported to be fertile [210].

#### 5.1.7. ZPB1/sp38/IAM38

ZPB1/sp38/IAM38 originated in spermatids and has been reported in mouse, human, and pig as well as bull spermatozoa. This protein is localized in the outer and inner acrosomal membrane or in the acrosomal matrix and is known as the secondary sperm-ZP binding receptor [234,235,236,237,238]. Nevertheless, ZPB1 was also detected on the surface of capacitated spermatozoa in boar as well as in bull and, due to its localization, may be implicated in the primary sperm contact with ZP [2,239].

#### 5.1.8. SPACA2/SP-10/ACV1

SPACA2/SP-10/ACV1 is another protein proposed as a sperm-ZP binding receptor that has been identified in the acrosomal matrix in all species mentioned above [80,241,242,243,244]. Nevertheless, the SPACA2 occurrence on the surface of capacitated boar spermatozoa implies a possible role in the primary attachment to ZP [80,241]. 

### 5.2. Mouse and Human Sperm-ZP Binding Receptors

This subsection is focused on molecules with ZP-binding affinity that are shared between human and mouse spermatozoa. These include α-1-3-fucosyltransferase, α-D-mannosidase, cysteine-rich secretory protein 1, zona receptor kinase, and fertilization antigen-1, all reviewed below.

#### 5.2.1. α-1-3-Fucosyltransferase (FUT5)

The α-1-3-fucosyltransferase (FUT5) was detected on the plasma membrane of both ejaculated and capacitated mouse spermatozoa [168,169]. Mouse FUT5 plays an important role in a variety of cell surface glycosylation events, mostly during sperm maturation. During spermatogenesis, FUT5 modulates germ cell-Sertoli cell interactions within the seminiferous epithelium; it may be involved in the adhesion of germ cells to the surrounding Sertoli cell and their release in the seminiferous tubule lumen during spermiation [168]. However, the presence of FUT5 activity on the surface of capacitated spermatozoa implies the involvement in ZP binding [169]. FUT5 was identified in the human spermatozoa, where it is an integral membrane protein localized to the acrosomal region, which is consistent with the proposed ZP-binding ability [170].

#### 5.2.2. α-D-Mannosidase (MAN2)

Another conserved enzyme with ZP-binding affinity is α-D-mannosidase [171,172,173]. It is an integral sperm plasma membrane protein that probably facilitates ZP binding by adhering to mannose content present on ZP oligosaccharide chains [171,172,173]. The participation of α-D-mannosidase in ZP binding was shown by Cornwall et al. [171] in the experiment where α-mannosidase inhibitor treatment led to the reduction in the number of bound spermatozoa to ZP.

#### 5.2.3. Cysteine-Rich Secretory Protein (CRISP1)

Cysteine-rich secretory protein, CRISP1, was identified in the mouse, rat, and human spermatozoa [174,175,178]. It is an epididymal protein that binds to the sperm head surface during epididymal transit [178]. CRISP1 is a multifunctional protein reported to participate in primary sperm-ZP binding [176] as well as in gamete fusion [177]. Studies performed by Da Ros et al. [297] showed that CRISP1 knockout spermatozoa exhibited an impaired ability to penetrate both ZP-intact and ZP-free oocytes that support the proposed roles of CRISP1 during gamete interaction.

#### 5.2.4. Zona Receptor Kinase (ZRK)

Zona receptor kinase (ZRK) is a 95 kDa protein localized in the acrosomal region of the sperm head surface in mice [180] and humans [181]. Binding of ZP3 to ZRK stimulates its kinase activity, while synthetic ZRK peptides inhibit sperm-ZP binding implying the role of ZRK in sperm-ZP binding [181].

#### 5.2.5. Fertilization Antigen-1 (FA-1)

Fertilization antigen-1 (FA-1) is a 23 kDa glycoprotein localized on the sperm surface, and similar to ZRK, it possesses a tyrosine kinase activity [298]. FA-1 is synthesized by male germ cells [299] and was suggested as the molecule mediating gamete recognition and the primary sperm-ZP binding in humans [182,184,185,186,188] and mouse models [183,187]. Anti-FA-1 antibodies significantly reduced human sperm-ZP binding [185,186].

#### 5.2.6. Angiotensin-Converting Enzyme 1 (ACE1)

Angiotensin-converting enzyme 1 (ACE1) has been proposed as a ZP-binding molecule due to its affinity for ZP [203]. Two forms of ACE1 are encoded by the same gene, namely the somatic ACE and germinal/testicular tACE (see reviews [300,301]). *ACE1*^−/−^ knock out mice were subfertile and showed reduced ZP binding, and fertility was rescued when the functional tACE gene was reintroduced [194,195]. tACE was also found on the human sperm surface [198]. Of note, ACE1 homolog ACE2 is expressed in male germ cells, Sertoli cells and Leydig cells [302,303] and was reported in boar seminal plasma as well [287]; however, its possible participation in sperm-ZP binding has not been reported to date. During the global COVID-19 pandemic, ACE2 is getting significant attention as the cellular receptor of the SARS-CoV-2 virus [304]. 

#### 5.2.7. P34H/Carbonyl Reductase/DCXR

In human spermatozoa, another molecule with ZP-binding affinity termed P34H/carbonyl reductase/DCXR has been reported [215,216]. The DCXR was initially reported in the hamster [211,212] and later in murine [214], bovine [218,219] and porcine spermatozoa [217]. It is a GPI-anchored epididymal secretory protein within the sperm plasma membrane overlying the acrosome, where it is incorporated during epididymal transit via epididymosomes [215,216,220,305]. Anti-DCXR antibody saturated spermatozoa displayed decreased binding to ZP in humans [215] and hamsters [213], but not mice [214].

#### 5.2.8. Other Human Sperm-ZP Binding Proteins

Lastly, for human spermatozoa, an effort was made to identify the respective sperm-ZP binding proteins by a combination of two approaches: (i) immunoblotting of human sperm extracts probed with anti-sperm antibodies from infertile men, and (ii) far western blotting of human sperm proteins overlayed with individual recombinant human (rh) ZP2, ZP3 and ZP4 proteins expressed in Chinese hamster ovary cells [306]. Nine different proteins were identified to bind rhZP2-4, namely PKM (PK3), ENO1, GADPH, ALDOA, TPI1 (glycolytic enzymes), GSTM, GPX4 (detoxifying enzymes), VDAC2 (ion transport), and ODF2 (sperm tail cytoskeleton). The acrosomal localization of some of the identified ZP-binding sperm proteins (ALDOA, GSTM, and ALDOA) was confirmed in said study. Furthermore, anti-ALDOA and anti-VDAC2 pre-incubated spermatozoa displayed reduced binding to zona-intact unfertilized human oocytes compared to the controls. GADPH and PKM (PK-S) were reported on the acrosome as well as in the flagellum in a separate study by Feiden et al. [307]. The other identified proteins require further studies, especially ODF2, a sperm tail protein. The authors Petit et al. [306] mention in the discussion that ODF2 localized on the sperm head by immunofluorescence; however, this still required plasma membrane permeabilization just as the flagellar detection of ODF2 would. We recently noticed the same pattern with another flagellar protein, CCDC39, that immunolocalized in the flagellum as well as in the very well defined apical portion of the head of boar spermatozoa only after methanol fixation/permeabilization (Zigo et al. unpublished).

### 5.3. Candidate Boar Sperm-ZP Receptors

#### 5.3.1. Spermadhesins

The most thoroughly studied molecules with ZP-binding affinity in the pig model are the seminal plasma-derived spermadhesins, the abundant sperm surface proteins that constitute the bulk of boar seminal plasma proteome [287,308,309,310,311]. Spermadhesins have multiple roles in porcine fertilization. Firstly, they stabilize the sperm plasma membrane [226] and participate in the formation of the oviductal reservoir [312], and secondly, they are decapacitating factors that prevent premature sperm capacitation after ejaculation and later mediate sperm adhesion to both the oviductal epithelial cells of the sperm reservoir and the oocyte zona [224,227,231]. Five proteins from the spermadhesin family and their differentially glycosylated isoforms were identified: PSP-I, PSP-II, AWN, AQN1, and AQN3. The main candidates implicated in sperm-ZP binding include AWN, AQN1 and AQN3. Their ZP-binding activity has been investigated using different approaches, such as a binding study on the blot, ZP-affinity chromatography, blocking of the sperm-ZP interaction with specific antibodies or a purified protein [217,222,223,224,225,226,227,228,229]. Spermadhesins belong to the protein family with a heparin-binding affinity [227]. Spermadhesins AWN, AQN1, and AQN3 identically bind to Galβ(1–3)-GalNAc and Ga1β(1–4)-GlcNAc carbohydrate structures of ZP glycoproteins [224,226]. The AQN1 associates with the sperm plasma membrane via an indirect lipid-binding mechanism (i.e., the binding via transmembrane proteins or proteins closely associated with membrane phospholipids). AWN and AQN-3 stabilize the plasma membrane over the acrosomal vesicle and are released from the surface during capacitation [224,226]. Spermadhesins AWN and AQN form complexes with another seminal plasma protein—DQH/BSP1/pB1 and bind the sperm surface [231]. Their deaggregation during sperm capacitation is regulated by the ubiquitin-proteasome system [292].

#### 5.3.2. DQH/BSP1/pB1

The DQH/BSP1 (a boar homolog to bull BSP1; binder of sperm (BSP) protein), a sperm surface protein [227] also known as pB1 [313], was described as a heparin-binding protein and localized on the surface of ejaculated boar spermatozoa [227,232]. This protein consists of the N-terminal O-glycosylated peptide followed by two fibronectin-type II repeats [314] and is homologous to the proteins abundantly present in bull seminal plasma [315] (for a BSP review, we recommend Plante et al. [316]). A monoclonal antibody against DQH reduced the binding of sperm to ZP, suggesting the role of DQH protein in the primary sperm-ZP binding [232].

#### 5.3.3. Other Boar Sperm-ZP Binding Proteins 

Several other boar sperm proteins with ZP-binding affinity were reported. Adhesion protein z (APz; a 55 kDa protein) has been obtained by affinity chromatography from sperm lysate. APz has been implicated in the adhesion of capacitated spermatozoa to the oocyte prior to the acrosomal exocytosis [251,252]. As noted previously, ZPBP1 that was originally described in the porcine sperm acrosome and inner acrosomal membrane [238,239,240], was reported to translocate to the surface during capacitation where it may participate in the primary sperm-ZP interactions [2,80,81]. Furthermore, *ZPBP1*^−/−^ knock out mice were found to be infertile due to improper compaction of acrosome during spermatogenesis [234]. Multiple ZP-binding proteins isolated from the apical sperm head plasma membranes were reported by van Gestel et al. [217], including ACRBP/acrosin binding protein, DCXR/carbonyl reductase, KCNC4/potassium voltage-gated channel PTPN13/protein tyrosine phosphatase, and PRDX5/peroxiredoxin 5, and ADAM2. Other ADAM family proteins were reported to have ZP-binding affinity and are believed to play a role in the primary sperm-ZP interactions; these are ADAM3 [317], ADAM5, and ADAM20-like [318]. Our group also reported several sperm surface proteins with the ZP-binding affinity that are highly likely to participate in primary sperm-ZP interactions; these include RAB2A, PKDREJ, as well as previously reported proteins ACE, MFGE8, and ACR [149,203].

### 5.4. Candidate Bull Sperm-ZP Receptors

Sperm-ZP binding receptors have not been investigated in detail in the bull. One of the proposed molecules that was reported to have ZP-binding affinity in the bull is carbonyl reductase DCXH/P25b. This protein is homologous with human P34H and rodent P26h and was discussed above.

Despite some abundant proteins of seminal plasma (such as PDC-109, also termed BSP-A1/A2) being present in bull spermatozoa [319], their connection with the sperm-ZP-binding activity has not been studied in detail. This seminal plasma protein has been ascribed a role in the formation of the oviductal sperm reservoir [320]. Nevertheless, the PDC-109 protein interaction network revealed its direct association with other proteins that regulate zona binding (SPAM1/PH-20/hyaluronidase, ACR, ZPBP1) [321]. Unlike the pig, bovine spermadhesins do not seem to participate in ZP binding [322]. A number of other proteins were identified from the bull sperm surface [2] as well as in bull seminal plasma [323] that are conserved between mammalian species and are thought to contribute to sperm-ZP binding. The function of these proteins in bovine fertilization is a subject for further investigation.

## 6. Lipid Microdomains and Multiprotein Complexes Implicated in Sperm-ZP Interaction

Although a substantial number of sperm molecules with ZP-binding affinity have been identified, the specific mechanism of the sperm-ZP interaction remains fairly unclear. The mechanistic model that was accepted for decades hypothesized that there was only one essential receptor for ZP on the sperm acrosomal surface, responsible for triggering the downstream signal transduction cascade. This simplistic “lock and key” theory was gradually disproved as various transgenic strains of KO mice lacking individual genes encoding presumptive ZP-binding proteins became available. An explanation to the question of why there are so many ZP-binding proteins was offered by Tanphaichitr et al. [80], as they reasoned that the circumstances under which these molecules were identified simply do not reflect in vivo situation. Rather than sperm-ZP binding being mediated by a single receptor-ligand interaction, multiple concomitants, perhaps synergistic binding events involved numerous sperm receptor species organized in distinct plasma membrane domains (reviewed in Redgrove et al. [324]).

Sperm capacitation is a process encompassing many dynamic changes in the protein composition of spermatozoa that ultimately leads to acquiring the full potential to bind to ZP and undergo acrosomal exocytosis. This protein reorganization during capacitation is initiated by cholesterol efflux that increases the plasma membrane fluidity and rearranges sperm surface proteins into lipid rafts that relocate and aggregate in the apical plasma membrane over the acrosome [81,325,326,327]. These aggregated sperm surface receptor domains serve as ZP-binding sites. As such, the multitude of sperm-ZP binding molecules is consistent with the presence of lipid rafts. Lipid rafts, also known as detergent-resistant membranes (DRMs) that are present in the outer leaflet of the plasma membrane bilayer, are enriched in cholesterol and sphingolipids [328]. Generally, DRMs are defined as small, heterogeneous, highly dynamic domains containing specific types of proteins and glycoproteins that serve to compartmentalize cellular processes such as signal transduction [81,329].

One of the major lipid components of sperm DRMs is sulfogalactosylglycerolipid (SGG) (reviewed in Tanphaichitr et al. [330,331]). The sperm SGG, also known as seminolipid, is an integral component of DRMs that is important during sperm raft formation via its interaction with cholesterol but also involved in sperm-ZP binding [326,332,333]. It has been proposed that SGG mediates the ZP binding via electrostatic interactions between sulfated galactosyl residues of SGG and glycoside moieties of ZP glycoproteins [334]. A sperm-ZP binding via SGG is also facilitated by its interaction with a raft-associated protein ARSA, discussed in the previous section.

**Figure 2:**
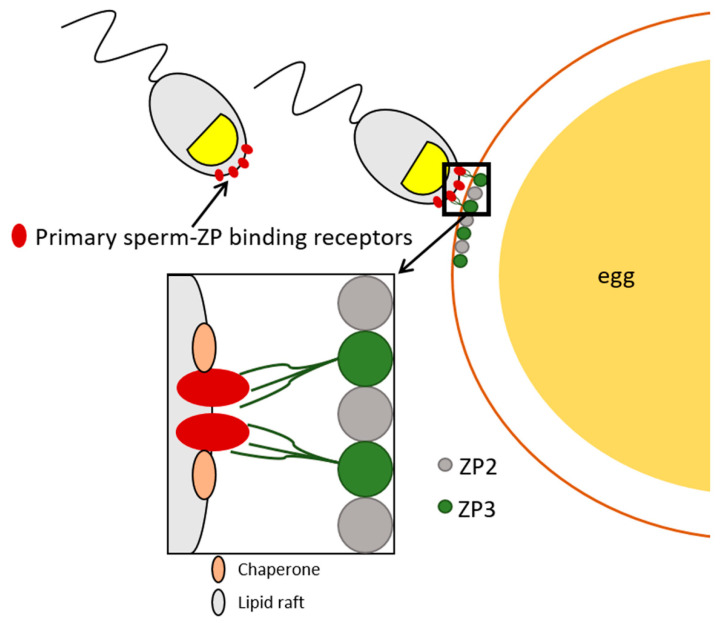
Illustration of primary sperm-ZP interaction. Sperm lipid rafts recognize sperm-binding molecules and transport them to the surface. This process is connected to the activation of chaperones that transport binding molecules into lipid raft microdomains, providing molecular machinery to assemble a receptor complex and subsequent competency of spermatozoa to bind to the ZP.

Other membrane-associated components that stabilize sperm-ZP-binding molecules and facilitate the remodeling and/or formation of sperm-ZP binding sites are molecular chaperones. Chaperones are generally crucial for proper protein folding, preventing protein aggregation, and maintaining protein homeostasis [335,336]. Several chaperones from the heat shock protein family, including HSP60, also termed chaperonin, Hsp70, HSP72, HSP90α and HSP90b1, also known as endoplasmin, have been identified on the sperm plasma membrane in mammalian species [80,254,337,338,339,340,341,342]. The surface localization of sperm chaperones increases substantially during capacitation while they are lost during the acrosomal exocytosis [343,344]. Furthermore, chaperones relocate to the periacrosomal region during capacitation while ushering ZP-binding molecules into lipid microdomains localized on the sperm surface [253,345]. These lipid microdomains may provide a favorable environment for chaperones to mediate the assembly of functional ZP-binding receptor complexes [346]. Sperm surface chaperones were found to play an indirect role in the sperm-ZP binding by stabilizing the functional ZP-binding receptors [253,345], see Figure 2, which agrees with the previous observation of the absence of chaperones leading to the reduction of sperm ability to bind the ZP [344]. 

Chaperones’ involvement in the incorporation of ZP-binding receptors into high-molecular-weight (HMW) complexes have been reported in mice [345], humans [253], and pigs [80,254]. Surprisingly, only a small number of sperm-ZP binding proteins were identified in the HMW complexes such as ZAN, ACR, ACRBP, ASPX, ZP3R or ZPBP1/ZPBP2 (all of the proteins of intra-acrosomal origin, as discussed previously), as well as seminal plasma derived MGFE8, tACE1, AQN3 and AWN [80,254]. The nature of the experimental approach including non-denaturing isolation as well as analysis of native blue PAGE, separated protein complexes, reflects the situation in vivo more accurately and might explain why there are so many seemingly redundant proteins with ZP-binding affinity. Beyond that, this particular approach allowed the identification of 26S proteasome being a part of the HMW complexes. This universal protein degrading and recycling holoenzyme was found vital to many aspects of mammalian fertilization [347,348] but did not possess ZP-binding ability; however, thanks to its presence in the acrosomal HMW complexes, the 26S proteasome can participate in ZP degradation during sperm-ZP penetration, as reported in mammals [291], birds [349], ascidians and echinoderms [350].

## 7. Conclusions

The primary sperm-ZP binding is an essential step in the mammalian fertilization process. Sperm interaction with ZP glycoproteins is a multimolecular event that requires the involvement of sperm surface receptors with complementary ZP carbohydrates. This interaction is not entirely species-specific in mammals, unlike the lower taxa with external fertilization that spawn in the water to reproduce. Primary sperm-ZP binding in vivo is likely mediated by the coordinated action of multiple sperm proteins, including ZP receptors, chaperone proteins, and 26S proteasomes assembled into HMW complexes where each of them plays a specific role during the ZP recognition and gamete interaction. The occurrence of HMW complexes on the sperm surface and their association with molecular machines such as chaperones and proteasomes within membrane lipid rafts may help to understand the underlying molecular mechanism of sperm-ZP binding. The existence of HMW complexes in vivo offers an explanation of the high redundancy of ZP-binding molecules. Further efforts are necessary to fully understand the molecular mechanisms of HMW complexes’ interactions with ZP. The research on sperm-ZP binding proteins benefits animal reproduction and human infertility therapy primarily by identifying candidate male fertility markers and regulatory mechanisms involved in gamete transport and fertilization. The understanding of the molecular basis of sperm-ZP binding may find applications in human assisted reproductive therapy, the use of which has been increasing steadily as childbearing age increases and more options and improvements are introduced in clinics. Similarly, animal breeding will be ameliorated by improvements in biomarker-based livestock semen quality control, preservation and distribution. Based on the study of binding receptors by means of specific antibodies or sperm selection kits could be performed to be of benefit in mammalian fertility diagnostics. Additionally, targeted blocking of sperm-ZP binding at the level of sperm proteins could translate into novel non-hormonal contraceptives, with early success stories already known in the field of wildlife management and pest control.

## Figures and Tables

**Table 1:** Summary of zona pellucida (ZP) glycoproteins in different mammalian species. ZP protein AA sequences were taken from the UniProtKB database, uniport.org and the sequence alignment was performed using BLAST^®^ software blast.ncbi.nlm.nih.gov/BlastAlign.cgi.

Mammalian Species	ZP Gene	ZP Protein	Molecular Weight (kDa)	Homology with				References
				Mouse	Human	Porcine	Bovine	
**Mouse**	*ZP1 (ZPB1)*	ZP1	200 (dimer)	-	68%	-	-	[21,22,23,24,25]
	*ZP2 (ZPA)*	ZP2	120	-	58%	55%	57%	
	*ZP3 (ZPC)*	ZP3	83	-	68%	66%	64%	
	*ZP4 (ZPB*/*ZPB2)*	not expressed	-	-	-	-	-	
**Human**	*ZP1 (ZPB1)*	ZP1	65	68%	-	-	-	[26,27,28,29]
	*ZP2 (ZPA)*	ZP2	120	58%	-	64%	67%	
	*ZP3 (ZPC)*	ZP3	58	68%	-	74%	72%	
	*ZP4 (ZPB*/*ZPB2)*	ZP4	65	-	-	68%	69%	
**Porcine**	*ZP1 (ZPB1)*	not expressed	-	-	-	-	-	[30,31,32,33,34,35,36,37,38]
	*ZP2 (ZPA)*	ZP2/PZPL	90	55%	64%	-	78%	
	*ZP3 (ZPC)*	ZP3/ZP3-β	55	66%	74%	-	84%	
	*ZP4 (ZPB*/*ZPB2)*	ZP4/ZP-α	55	-	68%	-	76%	
**Bovine**	*ZP1 (ZPB1)*	not expressed	-	-		-	-	[39,40,41]
	*ZP2 (ZPA)*	ZP2	76	57%	67%	78%	-	
	*ZP3 (ZPC)*	ZP3	47	64%	72%	84%	-	
	*ZP4 (ZPB*/*ZPB2)*	ZP4	68	-	69%	76%	-	

**Table 2:** A summary of proteins with ZP-binding affinity.

Protein with ZP-Binding Affinity	Species	Origin	Localization	Binding Activity	References
**β1,4-Galactosyltransferase (B4GALT1/GalTase)**	Mouse/rat	Male germ cells	Plasma membrane overlying the acrosome region	Binding to N-acetylglucosamine (GlcNAc) residues of ZP3, an inducer of AE via G-proteins, binds to terminal GlcNAc residues on O-linked oligosaccharides of ZP3	[119,120,121,122,123,124]
	Human		Unknown	Binding to ZP is assumed	[125]
	Boar		Anterior part of the sperm head, PM of the acrosome region, periacrosomal region of the sperm head	Binding to N-acetylglucosamine (GlcNAc) residues of ZP3 and/or ZP4; not necessary for sperm to bind ZP	[126,127]
	Bull		Anterior part of the sperm head, periacrosomal region of the sperm head		[126,128]
**Proacrosin/acrosin (ACR)**	Mouse/rat	Pachytene spermatocytes	Sperm acrosomal part	Binding non-enzymatically to ZP glycoproteins, mediating the secondary or tight binding of spermatozoa to the zona pellucida following the acrosome reaction	[129,130,131,132,133]
	Human		Acrosome, sperm surface in acrosomal cap	Binding to the solubilized ZP, interaction with mannose residues in ZP	[134,135,136,137,138,139,140,141]
	Boar	Spermatids	Inner acrosomal membrane and acrosome, sperm surface in acrosomal cap	High-affinity binding activity to sulfated oligosaccharide chains in ZP, secondary binding molecule; mediating or primary binding molecule, ZP-binding activity	[142,143,144,145,146,147,148,149]
	Bull	Spermatids	Acrosomal region		[150,151,152]
**Zonadhesin (ZAN)**	Mouse	Male germ cells	Outer acrosomal membrane and acrosomal matrix, a portion of ZAN translocates to the apical head region during sperm capacitation	Binding to the extracellular matrix of the oocyte, stimulation of tyrosine kinase activity leading to acrosomal exocytosis	[80,153,154]
	Human	Male germ cells	Membrane protein, apical head region, acrosome matrix	Binding to ZP3	[155,156]
	Boar	Germ cells—haploid spermatids	Transmembrane protein, apical head region in acrosome matrix	Binding to sulfated carbohydrates in ZP	[153,157,158]
	Bull	Male germ cells	Outer acrosomal membrane and acrosomal matrix	Binding to the extracellular matrix of the oocyte (assumed based on the other species)	[159]
**Arylsulfatase A (ARSA/AS-A/SLIP1)**	Mouse/rat	Male germ cells, epididymal fluid	Acrosomal matrix, sperm surface overlying acrosome	Binding ability to ZP sulfated glycans	[160,161,162,163]
	Human		Acrosomal matrix, sperm surface overlying acrosome	ZP binding	[164,165]
	Boar		Sperm head surface and acrosome, the head anterior region	Binding to sulfated sugar residues of the acidic ZP glycans present in ZP3α	[166]
	Bull		Convex ridge of the plasma membrane in the acrosomal part	ZP binding, assumed	[167]
**α1–3-Fucosyltransefrase (FUT5)**	Mouse	Male germ cells	Sperm head plasma membrane	Binding sites or receptor for ZP, sperm–oocyte recognition	[168,169]
	Human		Integral membrane protein in the acrosomal region	Interaction with solubilized human zona pellucida	[170]
**α-D-Mannosidase (MAN2)**	Mouse	Male germ cells	Plasma membrane overlying the acrosome	Binding molecule or receptor for ZP	[171]
	Human		Sperm plasma membrane	Role as a ligand for sperm-ZP recognition and binding, sperm surface α-D-mannosidase binds high mannose oligosaccharide units of ZP	[172,173]
**Cysteine-rich secretory protein (CRISP1)**	Mouse/rat	Epididymis	Dorsal region of the acrosome	ZP-binding activity	[174,175,176,177]
	Human	Epididymis	Sperm head plasma membrane?	Binding to ZP-intact human eggs, specific interaction with ZP3	[178,179]
**Zona receptor kinase (ZRK)**	Mouse		Sperm head plasma membrane	Binding to the extracellular matrix of the oocyte	[180]
	Human	Male germ cells	Sperm surface in the acrosomal region	Receptor for ZP3	[181]
**Fertilization antigen-1 (FA-1)**	Mouse	Testis	Sperm surface glycoprotein		[182,183,184,185,186,187,188]
	Human		Sperm surface glycoprotein	Recognition and binding to ZP3	[182,184,185,186,188]
**MFGE8/SED1/P47/lactadherin**	Mouse/rat	Male germ cells, Caput epididymis	Sperm plasma membrane overlying the acrosome	Recognition and binding to carbohydrate residues of mZP2 and mZP3	[189,190]
	Human		Sperm plasma membrane overlying the acrosome	ZP-binding activity, assumed	[191]
	Boar	Testis	Peripherally associated, the apical ridge of the sperm head or entire acrosome region	ZP-binding activity	[80,149,192,193]
**Angiotensin-converting enzyme 1 (ACE1)**	Mouse	Spermatids	Sperm plasma membrane overlying the acrosome	ZP-binding activity	[194,195]
	Human	Spermatids, Seminal plasma	Sperm plasma membrane overlying the acrosome, connecting piece, midpiece	ZP-binding activity, assumed	[196,197,198,199]
	Boar	Spermatids, epididymal fluid Seminal plasma	Sperm plasma membrane overlying the acrosome, connecting piece, midpiece	ZP-binding activity	[200,201,202,203]
	Bull	Spermatids, epididymal fluid Seminal plasma	Sperm plasma membrane overlying the acrosome, connecting piece, principal piece	ZP-binding activity, assumed	[201,202,203,204,205,206]
**ZP3R/sp56/AM67**	Mouse/rat/guinea pig	Male germ cells	Overlying the sperm acrosome, the head of acrosome intact sperm, plasma membrane protein	Binding to terminal galactose residue present on ZP3 O-linked oligosaccharides	[78,207,208,209,210]
**P26h/P34H/P25b/carbonyl reductase (DCXR)**	Mouse/hamster	Epididymis—epididymosomes	Plasma membrane overlying the acrosome		[211,212,213,214]
	Human	Epididymis—epididymosomes	Plasma membrane overlying the acrosome	Involved in the primary ZP binding	[215,216]
	Boar		Apical plasma membrane		[217]
	Bull	Epididymis—epididymosomes	Plasma membrane overlying the acrosome		[218,219,220]
**Spermadhesins AWN, AQN1, AQN3**	Boar	Seminal plasma	Sperm plasma membrane surface	Binding to Galβ(1–3)-GalNAc and Ga1β(1–4)-GlcNAc carbohydrate structures, ZP-binding activity	[217,221,222,223,224,225,226,227,228,229]
**Binder of sperm protein DQH/BSP1/pB1**	Boar	Seminal vesicles	Sperm plasma membrane surface, entire sperm head, in the acrosome region	Interaction with sialylated ZP glycoproteins	[230,231,232]
	Bull	Seminal vesicles		Nonreducing terminal α-mannosyl residues of the N-linked high-mannose-type chains	[108,114,233]
**ZPBP1/sp38/IAM38**	Mouse	Spermatids	Outer and inner acrosomal membrane	Secondary ZP binding	[234,235]
	Human	Spermatids	Acrosomal matrix	Secondary ZP binding	[236,237]
	Boar	Spermatids	Acrosomal matrix, inner acrosomal membrane, sperm surface in capacitated spermatozoa	Secondary ZP binding may be involved in primary ZP binding due to its localization in capacitated spermatozoa	[238,239,240]
	Bull	Spermatids	Acrosomal matrix, inner acrosomal membrane, sperm surface in capacitated spermatozoa	Secondary ZP binding may be involved in primary ZP binding due to its localization in capacitated spermatozoa	[2,240]
**SPACA2/SP-10/ACV1**	Mouse	Spermatids	Acrosomal matrix	Sperm attachment to ZP and ZP penetration was inhibited by anti-SP-10 antibodies	[241]
	Human	Spermatids	Acrosomal matrix	SP-10 does not seem to be involved in ZP binding; however, ZP penetration was inhibited by anti-SP-10 antibodies	[241,242,243]
	Boar	Spermatids	Acrosomal matrix, sperm surface in capacitated spermatozoa	Surface localization implies the role in primary ZP binding, sperm attachment to ZP and ZP penetration was inhibited by anti-SP-10 antibodies	[80,241]
	Bull	Spermatids	Acrosomal matrix	Anti-SP-10 antibodies reduced secondary sperm-ZP binding	[244]
**alpha-L-fucosidase (FUCA1)**	Mouse/Rat	Spermatids Seminal plasma	Plasma membrane overlying the acrosome, equatorial segment	Anti-FUCA1 antibodies inhibited ZP binging	[245,246,247]
	Human	Spermatids Seminal plasma	Plasma membrane overlying the acrosome, equatorial segment	ZP binding assumed	[248,249]
	Bull	Spermatids Seminal plasma	Unknown	ZP binding assumed	[250]
**Adhesion protein z (APz)**	Boar	Epididymis	Integral plasma membrane protein	Adhesion of capacitated sperm to the oocyte prior to the acrosomal reaction	[251,252]
**26S proteasome**	Human		Plasma membrane overlying the acrosome	Component of high-molecular-weight ZP-binding complexes	[253]
	Boar	Spermatids	Plasma membrane overlying the acrosome	Component of high-molecular-weight ZP-binding complexes	[254]

## Data Availability

Not applicable.

## References

[1] Brewis I.A., Gadella B.M. (2010). Sperm surface proteomics: From protein lists to biological function. Mol. Hum. Reprod..

[2] Byrne K., Leahy T., McCulloch R., Colgrave M.L., Holland M.K. (2012). Comprehensive mapping of the bull sperm surface proteome. Proteomics.

[3] Kasvandik S., Sillaste G., Velthut-Meikas A., Mikelsaar A.V., Hallap T., Padrik P., Tenson T., Jaakma U., Koks S., Salumets A. (2015). Bovine sperm plasma membrane proteomics through biotinylation and subcellular enrichment. Proteomics.

[4] Rickard J.P., de Graaf S.P. (2020). Sperm surface changes and their consequences for sperm transit through the female reproductive tract. Theriogenology.

[5] Yanagimachi R., Knobil E., Neill J.D. (1994). Mammalian fertilization. The Physiology of Reproduction.

[6] Gadella B.M., Boerke A. (2016). An update on post-ejaculatory remodeling of the sperm surface before mammalian fertilization. Theriogenology.

[7] Clark G.F. (2014). A role for carbohydrate recognition in mammalian sperm-egg binding. Biochem. Biophys. Res. Commun..

[8] Wassarman P.M. (2005). Contribution of mouse egg zona pellucida glycoproteins to gamete recognition during fertilization. J. Cell. Physiol..

[9] Rosano G., Caille A.M., Gallardo-Rios M., Munuce M.J. (2007). D-Mannose-binding sites are putative sperm determinants of human oocyte recognition and fertilization. Reprod. Biomed. Online.

[10] Sinowatz F., Wessa E., Neumüller C., Palma G. (2003). On the species specificity of sperm binding and sperm penetration of the zona pellucida. Reprod. Domest. Anim..

[11] Topfer-Petersen E., Ekhlasi-Hundrieser M., Tsolova M. (2008). Glycobiology of fertilization in the pig. Int. J. Dev. Biol..

[12] Takahashi K., Kikuchi K., Uchida Y., Kanai-Kitayama S., Suzuki R., Sato R., Toma K., Geshi M., Akagi S., Nakano M. (2013). Binding of Sperm to the Zona Pellucida Mediated by Sperm Carbohydrate-Binding Proteins is not Species-Specific in Vitro between Pigs and Cattle. Biomolecules.

[13] Wassarman P.M., Litscher E.S., Litscher E.S., Wassarman P.M. (2018). The mouse egg’s zona pellucida. Current Topics in Developmental Biology.

[14] Evans J.P. (2020). Preventing polyspermy in mammalian eggs-Contributions of the membrane block and other mechanisms. Mol. Reprod. Dev..

[15] Fahrenkamp E., Algarra B., Jovine L. (2020). Mammalian egg coat modifications and the block to polyspermy. Mol. Reprod. Dev..

[16] Harris J.D., Hibler D.W., Fontenot G.K., Hsu K.T., Yurewicz E.C., Sacco A.G. (1994). Cloning and characterization of zona pellucida genes and cDNAs from a variety of mammalian species: The ZPA, ZPB and ZPC gene families. DNA Seq..

[17] Spargo S.C., Hope R.M. (2003). Evolution and nomenclature of the zona pellucida gene family. Biol. Reprod..

[18] Goudet G., Mugnier S., Callebaut I., Monget P. (2008). Phylogenetic analysis and identification of pseudogenes reveal a progressive loss of zona pellucida genes during evolution of vertebrates. Biol. Reprod..

[19] Smith J., Paton I.R., Hughes D.C., Burt D.W. (2005). Isolation and mapping the chicken zona pellucida genes: An insight into the evolution of orthologous genes in different species. Mol. Reprod. Dev..

[20] Gupta S.K., Litscher E.S., Wassarman P.M. (2018). The human egg’s zona pellucida. Current Topics in Developmental Biology.

[21] Bokhove M., Jovine L. (2018). Structure of Zona Pellucida Module Proteins. Curr. Top. Dev. Biol..

[22] Conner S.J., Lefievre L., Hughes D.C., Barratt C.L. (2005). Cracking the egg: Increased complexity in the zona pellucida. Hum. Reprod..

[23] Greve J.M., Wassarman P.M. (1985). Mouse egg extracellular coat is a matrix of interconnected filaments possessing a structural repeat. J. Mol. Biol..

[24] Hughes D.C., Barratt C.L. (1999). Identification of the true human orthologue of the mouse Zp1 gene: Evidence for greater complexity in the mammalian zona pellucida?. Biochim. Biophys. Acta (BBA) Gene Struct. Expr..

[25] Wassarman P.M. (1988). Zona pellucida glycoproteins. Annu. Rev. Biochem..

[26] Chiu P.C., Wong B.S., Lee C.L., Pang R.T., Lee K.F., Sumitro S.B., Gupta S.K., Yeung W.S. (2008). Native human zona pellucida glycoproteins: Purification and binding properties. Hum. Reprod..

[27] Gupta S.K., Bhandari B., Shrestha A., Biswal B.K., Palaniappan C., Malhotra S.S., Gupta N. (2012). Mammalian zona pellucida glycoproteins: Structure and function during fertilization. Cell Tissue Res..

[28] Lefièvre L., Conner S.J., Salpekar A., Olufowobi O., Ashton P., Pavlovic B., Lenton W., Afnan M., Brewis I.A., Monk M. (2004). Four zona pellucida glycoproteins are expressed in the human. Hum. Reprod..

[29] Nishimura K., Dioguardi E., Nishio S., Villa A., Han L., Matsuda T., Jovine L. (2019). Molecular basis of egg coat cross-linking sheds light on ZP1-associated female infertility. Nat. Commun..

[30] Hasegawa A., Koyama K., Okazaki Y., Sugimoto M., Isojima S. (1994). Amino acid sequence of a porcine zona pellucida glycoprotein ZP4 determined by peptide mapping and cDNA cloning. J. Reprod. Fertil..

[31] Hedrick J.L., Wardrip N.J. (1986). Isolation of the zona pellucida and purification of its glycoprotein families from pig oocytes. Anal. Biochem..

[32] Hedrick J.L., Wardrip N.J. (1987). On the macromolecular composition of the zona pellucida from porcine oocytes. Dev. Biol..

[33] Nakano M., Hatanaka Y., Sawai T., Kobayashi N., Tobita T. (1987). Fractionation of glycoproteins from porcine zonae pellucidae into three families by high-performance liquid chromatography. Biochem. Int..

[34] Nakano M., Yonezawa N., Hatanaka Y., Noguchi S. (1996). Structure and function of the N-linked carbohydrate chains of pig zona pellucida glycoproteins. J. Reprod. Fertil. Suppl..

[35] Topfer-Petersen E., Mann K., Calvete J.J. (1993). Identification of porcine oocyte 55 kDa alpha and beta proteins within the zona pellucida glycoprotein families indicates that oocyte sperm receptor activity is associated with different zone pellucida proteins in different mammalian species. Biol. Chem..

[36] Wardrip N.J., Hedrick J.L. (1985). Pig zona pellucida 25K and 65K glycoproteins are derived from Hydrolysis and reduction of the 90K family. J. Cell Biol..

[37] Yurewicz E.C., Pack B.A., Sacco A.G. (1991). Isolation, composition, and biological activity of sugar chains of porcine oocyte zona pellucida 55K glycoproteins. Mol. Reprod. Dev..

[38] Yurewicz E.C., Sacco A.G., Subramanian M.G. (1987). Structural characterization of the Mr = 55,000 antigen (ZP3) of porcine oocyte zona pellucida. Purification and characterization of alpha- and beta-glycoproteins following digestion of lactosaminoglycan with endo-beta-galactosidase. J. Biol. Chem..

[39] Noguchi S., Yonezawa N., Katsumata T., Hashizume K., Kuwayama M., Hamano S., Watanabe S., Nakano M. (1994). Characterization of the zona pellucida glycoproteins from bovine ovarian and fertilized eggs. Biochim. Biophys. Acta (BBA) Gene Struct. Expr..

[40] Yonezawa N., Fukui N., Kuno M., Shinoda M., Goko S., Mitsui S., Nakano M. (2001). Molecular cloning of bovine zona pellucida glycoproteins ZPA and ZPB and analysis for sperm-binding component of the zona. Eur. J. Biochem..

[41] Yonezawa N., Kanai S., Nakano M. (2007). Structural significance of N-glycans of the zona pellucida on species-selective recognition of spermatozoa between pig and cattle. Soc. Reprod. Fertil. Suppl..

[42] Abou-Haila A., Bendahmane M., Tulsiani D.R. (2014). Significance of egg’s zona pellucida glycoproteins in sperm-egg interaction and fertilization. Minerva Ginecol..

[43] Yonezawa N. (2014). Posttranslational modifications of zona pellucida proteins. Adv. Exp. Med. Biol..

[44] Yonezawa N., Sawada H., Inoue N., Iwano M. (2014). Involvement of Carbohydrate Residues of the Zona Pellucida in In Vitro Sperm Recognition in Pigs and Cattle. Sexual Reproduction in Animals and Plants.

[45] Topfer-Petersen E. (1999). Carbohydrate-based interactions on the route of a spermatozoon to fertilization. Hum. Reprod. Update.

[46] Wassarman P.M., Litscher E.S. (2001). Towards the molecular basis of sperm and egg interaction during mammalian fertilization. Cells Tissues Organs.

[47] Hoodbhoy T., Dean J. (2004). Insights into the molecular basis of sperm-egg recognition in mammals. Reproduction.

[48] Shalgi R., Maymon R., Bar-Shira B., Amihai D., Skutelsky E. (1991). Distribution of lectin receptors sites in the zona pellucida of follicular and ovulated rat oocytes. Mol. Reprod. Dev..

[49] Maymon B.B., Maymon R., Ben-Nun I., Ghetler Y., Shalgi R., Skutelsky E. (1994). Distribution of carbohydrates in the zona pellucida of human oocytes. J. Reprod. Fertil..

[50] Parillo F., Stradaioli G., Dall’Aglio C., Verini-Supplizi A. (1996). Characterization of the complex carbohydrates in the zona pellucida of mammalian oocytes using lectin histochemistry. Vet. Res. Commun..

[51] Katsumata T., Noguchi S., Yonezawa N., Tanokura M., Nakano M. (1996). Structural characterization of the N-linked carbohydrate chains of the zona pellucida glycoproteins from bovine ovarian and fertilized eggs. Eur. J. Biochem..

[52] Lucas H., Bercegeay S., Le Pendu J., Jean M., Mirallie S., Barriere P. (1994). A fucose-containing epitope potentially involved in gamete interaction on the human zona pellucida. Hum. Reprod..

[53] Noguchi S., Hatanaka Y., Tobita T., Nakano M. (1992). Structural analysis of the N-linked carbohydrate chains of the 55-kDa glycoprotein family (PZP3) from porcine zona pellucida. Eur. J. Biochem..

[54] Noguchi S., Nakano M. (1992). Structure of the acidic N-linked carbohydrate chains of the 55-kDa glycoprotein family (PZP3) from porcine zona pellucida. Eur. J. Biochem..

[55] Mori E., Hedrick J.L., Wardrip N.J., Mori T., Takasaki S. (1998). Occurrence of reducing terminal N-acetylglucosamine 3-sulfate and fucosylated outer chains in acidic N-glycans of porcine zona pellucida glycoproteins. Glycoconj. J..

[56] Töpfer-Petersen E., Petrounkina A.M., Ekhlasi-Hundrieser M. (2000). Oocyte-sperm interactions. Anim. Reprod. Sci..

[57] Kudo K., Yonezawa N., Katsumata T., Aoki H., Nakano M. (1998). Localization of carbohydrate chains of pig sperm ligand in the glycoprotein ZPB of egg zona pellucida. Eur. J. Biochem..

[58] Easton R.L., Patankar M.S., Lattanzio F.A., Leaven T.H., Morris H.R., Clark G.F., Dell A. (2000). Structural analysis of murine zona pellucida glycans. Evidence for the expression of core 2-type O-glycans and the Sd(a) antigen. J. Biol. Chem..

[59] Noguchi S., Nakano M. (1993). Structural characterization of the N-linked carbohydrate chains from mouse zona pellucida glycoproteins ZP2 and ZP3. Biochim. Biophys. Acta (BBA) Gene Struct. Expr..

[60] Dell A., Chalabi S., Easton R.L., Haslam S.M., Sutton-Smith M., Patankar M.S., Lattanzio F., Panico M., Morris H.R., Clark G.F. (2003). Murine and human zona pellucida 3 derived from mouse eggs express identical O-glycans. Proc. Natl. Acad. Sci. USA.

[61] Boja E.S., Hoodbhoy T., Fales H.M., Dean J. (2003). Structural characterization of native mouse zona pellucida proteins using mass spectrometry. J. Biol. Chem..

[62] Jimenez-Movilla M., Aviles M., Gomez-Torres M.J., Fernandez-Colom P.J., Castells M.T., de Juan J., Romeu A., Ballesta J. (2004). Carbohydrate analysis of the zona pellucida and cortical granules of human oocytes by means of ultrastructural cytochemistry. Hum. Reprod..

[63] Pang P.C., Chiu P.C., Lee C.L., Chang L.Y., Panico M., Morris H.R., Haslam S.M., Khoo K.H., Clark G.F., Yeung W.S. (2011). Human sperm binding is mediated by the sialyl-Lewis(x) oligosaccharide on the zona pellucida. Science.

[64] Yonezawa N., Mitsui S., Kudo K., Nakano M. (1997). Identification of an N-glycosylated region of pig zona pellucida glycoprotein ZPB that is involved in sperm binding. Eur. J. Biochem..

[65] Nakano M., Yonezawa N. (2001). Localization of sperm ligand carbohydrate chains in pig zona pellucida glycoproteins. Cells Tissues Organs.

[66] Von Witzendorff D., Maass K., Pich A., Ebeling S., Kölle S., Kochel C., Ekhlasi-Hundrieser M., Geyer H., Geyer R., Töpfer-Petersen E. (2009). Characterization of the acidic N-linked glycans of the zona pellucida of prepuberal pigs by a mass spectrometric approach. Carbohydr. Res..

[67] Hokke C.H., Damm J.B., Penninkhof B., Aitken R.J., Kamerling J.P., Vliegenthart J.F. (1994). Structure of the O-linked carbohydrate chains of porcine zona pellucida glycoproteins. Eur. J. Biochem..

[68] Lay K.M., Nakada T., Tatemoto H. (2013). Involvement of N-glycosylation of zona glycoproteins during meiotic maturation in sperm-zona pellucida interactions of porcine denuded oocytes. Anim. Sci. J..

[69] Clark G.F., Zimmerman S., Lafrenz D.E., Yi Y.J., Sutovsky P. (2010). Carbohydrate-mediated binding and induction of acrosomal exocytosis in a boar sperm-somatic cell adhesion model. Biol. Reprod..

[70] Suzuki K., Tatebe N., Kojima S., Hamano A., Orita M., Yonezawa N. (2015). The Hinge Region of Bovine Zona Pellucida Glycoprotein ZP3 Is Involved in the Formation of the Sperm-Binding Active ZP3/ZP4 Complex. Biomolecules.

[71] Ikeda K., Yonezawa N., Naoi K., Katsumata T., Hamano S., Nakano M. (2002). Localization of N-linked carbohydrate chains in glycoprotein ZPA of the bovine egg zona pellucida. Eur. J. Biochem..

[72] Florman H.M., Fissore R.A., Plant T.M., Zeleznik A.J. (2015). Fertilization in Mammals. Knobil and Neill’s Physiology of Reproduction.

[73] Georgadaki K., Khoury N., Spandidos D.A., Zoumpourlis V. (2016). The molecular basis of fertilization (Review). Int. J. Mol. Med..

[74] Okabe M. (2018). Sperm-egg interaction and fertilization: Past, present, and future. Biol. Reprod..

[75] Zigo M., Manaskova-Postlerova P., Zuidema D., Kerns K., Jonakova V., Tumova L., Bubenickova F., Sutovsky P. (2020). Porcine model for the study of sperm capacitation, fertilization and male fertility. Cell Tissue Res..

[76] Tanphaichitr N., Carmona E., Bou Khalil M., Xu H., Berger T., Gerton G.L. (2007). New insights into sperm-zona pellucida interaction: Involvement of sperm lipid rafts. Front. Biosci..

[77] Fraser L.R. (1987). Minimum and maximum extracellular Ca^2+^ requirements during mouse sperm capacitation and fertilization in vitro. J. Reprod. Fertil..

[78] Kim K.S., Gerton G.L. (2003). Differential release of soluble and matrix components: Evidence for intermediate states of secretion during spontaneous acrosomal exocytosis in mouse sperm. Dev. Biol..

[79] Wassarman P.M. (2009). Mammalian fertilization: The strange case of sperm protein 56. Bioessays.

[80] Tanphaichitr N., Kongmanas K., Kruevaisayawan H., Saewu A., Sugeng C., Fernandes J., Souda P., Angel J.B., Faull K.F., Aitken R.J. (2015). Remodeling of the plasma membrane in preparation for sperm-egg recognition: Roles of acrosomal proteins. Asian J. Androl..

[81] López-Salguero J.B., Fierro R., Michalski J.C., Jiménez-Morales I., Lefebvre T., Mondragón-Payne O., Baldini S.F., Vercoutter-Edouart A.S., González-Márquez H. (2020). Identification of lipid raft glycoproteins obtained from boar spermatozoa. Glycoconj. J..

[82] Bleil J.D., Wassarman P.M. (1980). Mammalian sperm-egg interaction: Identification of a glycoprotein in mouse egg zonae pellucidae possessing receptor activity for sperm. Cell.

[83] Bleil J.D., Wassarman P.M. (1983). Sperm-egg interactions in the mouse: Sequence of events and induction of the acrosome reaction by a zona pellucida glycoprotein. Dev. Biol..

[84] Beebe S.J., Leyton L., Burks D., Ishikawa M., Fuerst T., Dean J., Saling P. (1992). Recombinant mouse ZP3 inhibits sperm binding and induces the acrosome reaction. Dev. Biol..

[85] Bleil J.D., Wassarman P.M. (1988). Galactose at the nonreducing terminus of O-linked oligosaccharides of mouse egg zona pellucida glycoprotein ZP3 is essential for the glycoprotein’s sperm receptor activity. Proc. Natl. Acad. Sci. USA.

[86] Litscher E.S., Wassarman P.M. (1996). Characterization of mouse ZP3-derived glycopeptide, gp55, that exhibits sperm receptor and acrosome reaction-inducing activity in vitro. Biochemistry.

[87] Kinloch R.A., Sakai Y., Wassarman P.M. (1995). Mapping the mouse ZP3 combining site for sperm by exon swapping and site-directed mutagenesis. Proc. Natl. Acad. Sci. USA.

[88] Thall A.D., Malý P., Lowe J.B. (1995). Oocyte Gal alpha 1,3Gal epitopes implicated in sperm adhesion to the zona pellucida glycoprotein ZP3 are not required for fertilization in the mouse. J. Biol. Chem..

[89] Litscher E.S., Juntunen K., Seppo A., Penttilä L., Niemelä R., Renkonen O., Wassarman P.M. (1995). Oligosaccharide constructs with defined structures that inhibit binding of mouse sperm to unfertilized eggs in vitro. Biochemistry.

[90] Mori E., Mori T., Takasaki S. (1997). Binding of mouse sperm to beta-galactose residues on egg zona pellucida and asialofetuin-coupled beads. Biochem. Biophys. Res. Commun..

[91] Johnston D.S., Wright W.W., Shaper J.H., Hokke C.H., Van den Eijnden D.H., Joziasse D.H. (1998). Murine sperm-zona binding, a fucosyl residue is required for a high affinity sperm-binding ligand. A second site on sperm binds a nonfucosylated, beta-galactosyl-capped oligosaccharide. J. Biol. Chem..

[92] Bleil J.D., Greve J.M., Wassarman P.M. (1988). Identification of a secondary sperm receptor in the mouse egg zona pellucida: Role in maintenance of binding of acrosome-reacted sperm to eggs. Dev. Biol..

[93] Avella M.A., Baibakov B., Dean J. (2014). A single domain of the ZP2 zona pellucida protein mediates gamete recognition in mice and humans. J. Cell Biol..

[94] Saling P.M., Sowinski J., Storey B.T. (1979). An ultrastructural study of epididymal mouse spermatozoa binding to zonae pellucidae in vitro: Sequential relationship to the acrosome reaction. J. Exp. Zool..

[95] Saling P.M., Storey B.T. (1979). Mouse gamete interactions during fertilization in vitro. Chlortetracycline as a fluorescent probe for the mouse sperm acrosome reaction. J. Cell Biol..

[96] Baibakov B., Gauthier L., Talbot P., Rankin T.L., Dean J. (2007). Sperm binding to the zona pellucida is not sufficient to induce acrosome exocytosis. Development.

[97] Jin M., Fujiwara E., Kakiuchi Y., Okabe M., Satouh Y., Baba S.A., Chiba K., Hirohashi N. (2011). Most fertilizing mouse spermatozoa begin their acrosome reaction before contact with the zona pellucida during in vitro fertilization. Proc. Natl. Acad. Sci. USA.

[98] Inoue N., Satouh Y., Ikawa M., Okabe M., Yanagimachi R. (2011). Acrosome-reacted mouse spermatozoa recovered from the perivitelline space can fertilize other eggs. Proc. Natl. Acad. Sci. USA.

[99] Buffone M.G., Hirohashi N., Gerton G.L. (2014). Unresolved questions concerning mammalian sperm acrosomal exocytosis. Biol. Reprod..

[100] Foster J.A., Gerton G.L. (2016). The Acrosomal Matrix. Adv. Anat. Embryol. Cell Biol..

[101] Hirohashi N., Yanagimachi R. (2018). Sperm acrosome reaction: Its site and role in fertilization. Biol. Reprod..

[102] Clark G.F., Dell A. (2006). Molecular models for murine sperm-egg binding. J. Biol. Chem..

[103] Clark G.F. (2011). Molecular models for mouse sperm-oocyte binding. Glycobiology.

[104] Chakravarty S., Kadunganattil S., Bansal P., Sharma R.K., Gupta S.K. (2008). Relevance of glycosylation of human zona pellucida glycoproteins for their binding to capacitated human spermatozoa and subsequent induction of acrosomal exocytosis. Mol. Reprod. Dev..

[105] Ozgur K., Patankar M.S., Oehninger S., Clark G.F. (1998). Direct evidence for the involvement of carbohydrate sequences in human sperm-zona pellucida binding. Mol. Hum. Reprod..

[106] Baibakov B., Boggs N.A., Yauger B., Baibakov G., Dean J. (2012). Human sperm bind to the N-terminal domain of ZP2 in humanized zonae pellucidae in transgenic mice. J. Cell Biol..

[107] Yurewicz E.C., Sacco A.G., Gupta S.K., Xu N., Gage D.A. (1998). Hetero-oligomerization-dependent binding of pig oocyte zona pellucida glycoproteins ZPB and ZPC to boar sperm membrane vesicles. J. Biol. Chem..

[108] Yonezawa N., Kudo K., Terauchi H., Kanai S., Yoda N., Tanokura M., Ito K., Miura K., Katsumata T., Nakano M. (2005). Recombinant porcine zona pellucida glycoproteins expressed in Sf9 cells bind to bovine sperm but not to porcine sperm. J. Biol. Chem..

[109] Yonezawa N., Amari S., Takahashi K., Ikeda K., Imai F.L., Kanai S., Kikuchi K., Nakano M. (2005). Participation of the nonreducing terminal beta-galactosyl residues of the neutral N-linked carbohydrate chains of porcine zona pellucida glycoproteins in sperm-egg binding. Mol. Reprod. Dev..

[110] Sutton-Smith M., Wong N.K., Khoo K.H., Wu S.W., Yu S.Y., Patankar M.S., Easton R., Lattanzio F.A., Morris H.R., Dell A. (2007). Analysis of protein-linked glycosylation in a sperm-somatic cell adhesion system. Glycobiology.

[111] Berger T., Turner K.O., Meizel S., Hedrick J.L. (1989). Zona pellucida-induced acrosome reaction in boar sperm. Biol. Reprod..

[112] Mattioli M., Lucidi P., Barboni B. (1998). Expanded cumuli induce acrosome reaction in boar sperm. Mol. Reprod. Dev..

[113] Kanai S., Yonezawa N., Ishii Y., Tanokura M., Nakano M. (2007). Recombinant bovine zona pellucida glycoproteins ZP3 and ZP4 coexpressed in Sf9 cells form a sperm-binding active hetero-complex. FEBS J..

[114] Amari S., Yonezawa N., Mitsui S., Katsumata T., Hamano S., Kuwayama M., Hashimoto Y., Suzuki A., Takeda Y., Nakano M. (2001). Essential role of the nonreducing terminal alpha-mannosyl residues of the N-linked carbohydrate chain of bovine zona pellucida glycoproteins in sperm-egg binding. Mol. Reprod. Dev..

[115] Velásquez J.G., Canovas S., Barajas P., Marcos J., Jiménez-Movilla M., Gallego R.G., Ballesta J., Avilés M., Coy P. (2007). Role of sialic acid in bovine sperm-zona pellucida binding. Mol. Reprod. Dev..

[116] Florman H.M., First N.L. (1988). The regulation of acrosomal exocytosis. I. Sperm capacitation is required for the induction of acrosome reactions by the bovine zona pellucida in vitro. Dev. Biol..

[117] Herz Z., Northey D., Lawyer M., First N.L. (1985). Acrosome reaction of bovine spermatozoa in vivo: Sites and effects of stages of the estrous cycle. Biol. Reprod..

[118] Didion B.A., Graves C.N. (1986). In vivo capacitation and acrosome reaction of bovine sperm in estrous and diestrous cows. J. Anim. Sci..

[119] Shur B.D., Hall N.G. (1982). A role for mouse sperm surface galactosyltransferase in sperm binding to the egg zona pellucida. J. Cell Biol..

[120] Scully N.F., Shaper J.H., Shur B.D. (1987). Spatial and temporal expression of cell surface galactosyltransferase during mouse spermatogenesis and epididymal maturation. Dev. Biol..

[121] Miller D.J., Macek M.B., Shur B.D. (1992). Complementarity between sperm surface beta-1,4-galactosyltransferase and egg-coat ZP3 mediates sperm-egg binding. Nature.

[122] Shur B.D. (1993). Glycosyltransferases as cell adhesion molecules. Curr. Opin. Cell Biol..

[123] Gong X., Dubois D.H., Miller D.J., Shur B.D. (1995). Activation of a G protein complex by aggregation of beta-1,4-galactosyltransferase on the surface of sperm. Science.

[124] Shi X., Amindari S., Paruchuru K., Skalla D., Burkin H., Shur B.D., Miller D.J. (2001). Cell surface beta-1,4-galactosyltransferase-I activates G protein-dependent exocytotic signaling. Development.

[125] Huszar G., Sbracia M., Vigue L., Miller D.J., Shur B.D. (1997). Sperm plasma membrane remodeling during spermiogenetic maturation in men: Relationship among plasma membrane beta 1,4-galactosyltransferase, cytoplasmic creatine phosphokinase, and creatine phosphokinase isoform ratios. Biol. Reprod..

[126] Larson J.L., Miller D.J. (1997). Sperm from a variety of mammalian species express beta1,4-galactosyltransferase on their surface. Biol. Reprod..

[127] Rebeiz M., Miller D.J. (1999). Porcine sperm surface beta1,4galactosyltransferase binds to the zona pellucida but is not necessary or sufficient to mediate sperm-zona pellucida binding. Mol. Reprod. Dev..

[128] Tengowski M.W., Wassler M.J., Shur B.D., Schatten G. (2001). Subcellular localization of beta1,4-galactosyltransferase on bull sperm and its function during sperm-egg interactions. Mol. Reprod. Dev..

[129] Kallajoki M., Parvinen M., Suominen J.J. (1986). Expression of acrosin during mouse spermatogenesis: A biochemical and immunocytochemical analysis by a monoclonal antibody C 11 H. Biol. Reprod..

[130] Kashiwabara S., Baba T., Takada M., Watanabe K., Yano Y., Arai Y. (1990). Primary structure of mouse proacrosin deduced from the cDNA sequence and its gene expression during spermatogenesis. J. Biochem..

[131] Klemm U., Maier W.M., Tsaousidou S., Adham I.M., Willison K., Engel W. (1990). Mouse preproacrosin: cDNA sequence, primary structure and postmeiotic expression in spermatogenesis. Differentiation.

[132] Kremling H., Keime S., Wilhelm K., Adham I.M., Hameister H., Engel W. (1991). Mouse proacrosin gene: Nucleotide sequence, diploid expression, and chromosomal localization. Genomics.

[133] Watanabe K., Baba T., Kashiwabara S., Okamoto A., Arai Y. (1991). Structure and organization of the mouse acrosin gene. J. Biochem..

[134] Gilboa E., Elkana Y., Rigbi M. (1973). Purification and properties of human acrosin. Eur. J. Biochem..

[135] Schleuning W.D., Hell R., Fritz H. (1976). Multiple forms of human acrosin: Isolation and properties. Hoppe-Seyler’s Z. Physiol. Chem..

[136] Anderson R.A., Beyler S.A., Mack S.R., Zaneveld L.J. (1981). Characterization of a high-molecular-weight form of human acrosin. Comparison with human pancreatic trypsin. Biochem. J..

[137] Tesarik J., Drahorad J., Peknicova J. (1988). Subcellular immunochemical localization of acrosin in human spermatozoa during the acrosome reaction and zona pellucida penetration. Fertil. Steril..

[138] Kobayashi T., Matsuda Y., Oshio S., Kaneko S., Nozawa S., Mhori H., Akihama S., Fujimoto Y. (1991). Human acrosin: Purification and some properties. Arch. Androl..

[139] Moreno R.D., Sepúlveda M.S., de Ioannes A., Barros C. (1998). The polysulphate binding domain of human proacrosin/acrosin is involved in both the enzyme activation and spermatozoa-zona pellucida interaction. Zygote.

[140] Furlong L.I., Hellman U., Krimer A., Tezón J.G., Charreau E.H., Vazquez-Levin M.H. (2000). Expression of human proacrosin in Escherichia coli and binding to zona pellucida. Biol. Reprod..

[141] Furlong L.I., Veaute C., Vazquez-Levin M.H. (2005). Binding of recombinant human proacrosin/acrosin to zona pellucida glycoproteins. II. Participation of mannose residues in the interaction. Fertil. Steril..

[142] Schill W.B. (1975). Immunofluorescent localization of acrosin in spermatozoa by boar acrosin antibodies. Naturwissenschaften.

[143] Jones R., Brown C.R. (1987). Identification of a zona-binding protein from boar spermatozoa as proacrosin. Exp. Cell Res..

[144] Jones R., Brown C.R., Lancaster R.T. (1988). Carbohydrate-binding properties of boar sperm proacrosin and assessment of its role in sperm-egg recognition and adhesion during fertilization. Development.

[145] Baba T., Kashiwabara S., Watanabe K., Itoh H., Michikawa Y., Kimura K., Takada M., Fukamizu A., Arai Y. (1989). Activation and maturation mechanisms of boar acrosin zymogen based on the deduced primary structure. J. Biol. Chem..

[146] Baba T., Michikawa Y., Kawakura K., Arai Y. (1989). Activation of boar proacrosin is effected by processing at both N- and C-terminal portions of the zymogen molecule. FEBS Lett..

[147] Jones R. (1991). Interaction of zona pellucida glycoproteins, sulphated carbohydrates and synthetic polymers with proacrosin, the putative egg-binding protein from mammalian spermatozoa. Development.

[148] Puigmulé M., Fàbrega A., Yeste M., Bonet S., Pinart E. (2011). Study of the proacrosin-acrosin system in epididymal, ejaculated and in vitro capacitated boar spermatozoa. Reprod. Fertil. Dev..

[149] Zigo M., Dorosh A., Pohlova A., Jonakova V., Sulc M., Manaskova-Postlerova P. (2015). Panel of monoclonal antibodies to sperm surface proteins as a tool for monitoring localization and identification of sperm-zona pellucida receptors. Cell Tissue Res..

[150] Garner D.L., Easton M.P., Munson M.E., Doane M.A. (1975). Immunofluorescent localization of bovine acrosin. J. Exp. Zool..

[151] Mansouri A., Phi-van L., Geithe H.P., Engel W. (1983). Proacrosin/acrosin activity during spermiohistogenesis of the bull. Differentiation.

[152] De los Reyes M., Barros C. (2000). Immunolocalization of proacrosin/acrosin in bovine sperm and sperm penetration through the zona pellucida. Anim. Reprod. Sci..

[153] Gao Z., Garbers D.L. (1998). Species diversity in the structure of zonadhesin, a sperm-specific membrane protein containing multiple cell adhesion molecule-like domains. J. Biol. Chem..

[154] Tardif S., Wilson M.D., Wagner R., Hunt P., Gertsenstein M., Nagy A., Lobe C., Koop B.F., Hardy D.M. (2010). Zonadhesin is essential for species specificity of sperm adhesion to the egg zona pellucida. J. Biol. Chem..

[155] Gao Z., Harumi T., Garbers D.L. (1997). Chromosome localization of the mouse zonadhesin gene and the human zonadhesin gene (ZAN). Genomics.

[156] Wilson M.D., Riemer C., Martindale D.W., Schnupf P., Boright A.P., Cheung T.L., Hardy D.M., Schwartz S., Scherer S.W., Tsui L.C. (2001). Comparative analysis of the gene-dense ACHE/TFR2 region on human chromosome 7q22 with the orthologous region on mouse chromosome 5. Nucleic Acids Res..

[157] Hardy D.M., Garbers D.L. (1995). A sperm membrane protein that binds in a species-specific manner to the egg extracellular matrix is homologous to von Willebrand factor. J. Biol. Chem..

[158] Bi M., Hickox J.R., Winfrey V.P., Olson G.E., Hardy D.M. (2003). Processing, localization and binding activity of zonadhesin suggest a function in sperm adhesion to the zona pellucida during exocytosis of the acrosome. Biochem. J..

[159] Bi M. (2002). Biochemical and Functional Characterization of Zonadhesin: A Sperm Protein Potentially Mediating Species-Specific Sperm-Egg Adhesion during Fertilization. Ph.D. Thesis.

[160] Tanphaichitr N., Smith J., Mongkolsirikieart S., Gradil C., Lingwood C.A. (1993). Role of a gamete-specific sulfoglycolipid immobilizing protein on mouse sperm-egg binding. Dev. Biol..

[161] Moase C.E., Kamolvarin N., Kan F.W., Tanphaichitr N. (1997). Localization and role of sulfoglycolipid immobilizing protein 1 on the mouse sperm head. Mol. Reprod. Dev..

[162] Tantibhedhyangkul J., Weerachatyanukul W., Carmona E., Xu H., Anupriwan A., Michaud D., Tanphaichitr N. (2002). Role of sperm surface arylsulfatase A in mouse sperm-zona pellucida binding. Biol. Reprod..

[163] Ngernsoungnern A., Weerachatyanukul W., Saewu A., Thitilertdecha S., Sobhon P., Sretarugsa P. (2004). Rat sperm AS-A: Subcellular localization in testis and epididymis and surface distribution in epididymal sperm. Cell Tissue Res..

[164] Redgrove K.A., Nixon B., Baker M.A., Hetherington L., Baker G., Liu D.Y., Aitken R.J. (2012). The molecular chaperone HSPA2 plays a key role in regulating the expression of sperm surface receptors that mediate sperm-egg recognition. PLoS ONE.

[165] Bromfield E.G., Aitken R.J., Anderson A.L., McLaughlin E.A., Nixon B. (2015). The impact of oxidative stress on chaperone-mediated human sperm-egg interaction. Hum. Reprod..

[166] Carmona E., Weerachatyanukul W., Soboloff T., Fluharty A.L., White D., Promdee L., Ekker M., Berger T., Buhr M., Tanphaichitr N. (2002). Arylsulfatase a is present on the pig sperm surface and is involved in sperm-zona pellucida binding. Dev. Biol..

[167] Kelsey K.M., Zigo M., Thompson W.E., Kerns K., Manandhar G., Sutovsky M., Sutovsky P. (2020). Reciprocal surface expression of arylsulfatase A and ubiquitin in normal and defective mammalian spermatozoa. Cell Tissue Res..

[168] Cardullo R.A., Armant D.R., Millette C.F. (1989). Characterization of fucosyltransferase activity during mouse spermatogenesis: Evidence for a cell surface fucosyltransferase. Biochemistry.

[169] Ram P.A., Cardullo R.A., Millette C.F. (1989). Expression and topographical localization of cell surface fucosyltransferase activity during epididymal sperm maturation in the mouse. Gamete Res..

[170] Chiu P.C., Chung M.K., Koistinen R., Koistinen H., Seppala M., Ho P.C., Ng E.H., Lee K.F., Yeung W.S. (2007). Glycodelin-A interacts with fucosyltransferase on human sperm plasma membrane to inhibit spermatozoa-zona pellucida binding. J. Cell Sci..

[171] Cornwall G.A., Tulsiani D.R., Orgebin-Crist M.C. (1991). Inhibition of the mouse sperm surface alpha-D-mannosidase inhibits sperm-egg binding in vitro. Biol. Reprod..

[172] Tulsiani D.R., Skudlarek M.D., Orgebin-Crist M.C. (1990). Human sperm plasma membranes possess alpha-D-mannosidase activity but no galactosyltransferase activity. Biol. Reprod..

[173] Tesarik J., Mendoza C., Carreras A. (1991). Expression of D-mannose binding sites on human spermatozoa: Comparison of fertile donors and infertile patients. Fertil. Steril..

[174] Eberspaecher U., Roosterman D., Krätzschmar J., Haendler B., Habenicht U.F., Becker A., Quensel C., Petri T., Schleuning W.D., Donner P. (1995). Mouse androgen-dependent epididymal glycoprotein CRISP-1 (DE/AEG): Isolation, biochemical characterization, and expression in recombinant form. Mol. Reprod. Dev..

[175] Cohen D.J., Ellerman D.A., Cuasnicu P.S. (2000). Mammalian sperm-egg fusion: Evidence that epididymal protein DE plays a role in mouse gamete fusion. Biol. Reprod..

[176] Busso D., Cohen D.J., Maldera J.A., Dematteis A., Cuasnicu P.S. (2007). A novel function for CRISP1 in rodent fertilization: Involvement in sperm-zona pellucida interaction. Biol. Reprod..

[177] Cohen D.J., Maldera J.A., Vasen G., Ernesto J.I., Muñoz M.W., Battistone M.A., Cuasnicú P.S. (2011). Epididymal protein CRISP1 plays different roles during the fertilization process. J. Androl..

[178] Hayashi M., Fujimoto S., Takano H., Ushiki T., Abe K., Ishikura H., Yoshida M.C., Kirchhoff C., Ishibashi T., Kasahara M. (1996). Characterization of a human glycoprotein with a potential role in sperm-egg fusion: cDNA cloning, immunohistochemical localization, and chromosomal assignment of the gene (AEGL1). Genomics.

[179] Maldera J.A., Weigel Muñoz M., Chirinos M., Busso D., Ge Raffo F., Battistone M.A., Blaquier J.A., Larrea F., Cuasnicu P.S. (2014). Human fertilization: Epididymal hCRISP1 mediates sperm-zona pellucida binding through its interaction with ZP3. Mol. Hum. Reprod..

[180] Leyton L., Saling P. (1989). 95 kd sperm proteins bind ZP3 and serve as tyrosine kinase substrates in response to zona binding. Cell.

[181] Burks D.J., Carballada R., Moore H.D., Saling P.M. (1995). Interaction of a tyrosine kinase from human sperm with the zona pellucida at fertilization. Science.

[182] Naz R.K., Alexander N.J., Isahakia M., Hamilton M.S. (1984). Monoclonal antibody to a human germ cell membrane glycoprotein that inhibits fertilization. Science.

[183] Naz R.K., Phillips T.M., Rosenblum B.B. (1986). Characterization of the fertilization antigen 1 for the development of a contraceptive vaccine. Proc. Natl. Acad. Sci. USA.

[184] Naz R.K., Sacco A.G., Yurewicz E.C. (1991). Human spermatozoal FA-1 binds with ZP3 of porcine zona pellucida. J. Reprod. Immunol..

[185] Naz R.K., Brazil C., Overstreet J.W. (1992). Effects of antibodies to sperm surface fertilization antigen-1 on human sperm-zona pellucida interaction. Fertil. Steril..

[186] Kadam A.L., Fateh M., Naz R.K. (1995). Fertilization antigen (FA-1) completely blocks human sperm binding to human zona pellucida: FA-1 antigen may be a sperm receptor for zona pellucida in humans. J. Reprod. Immunol..

[187] Zhu X., Naz R.K. (1997). Fertilization antigen-1: cDNA cloning, testis-specific expression, and immunocontraceptive effects. Proc. Natl. Acad. Sci. USA.

[188] Naz R.K., Zhu X. (2002). Molecular cloning and sequencing of cDNA encoding for human FA-1 antigen. Mol. Reprod. Dev..

[189] Ensslin M.A., Shur B.D. (2003). Identification of mouse sperm SED1, a bimotif EGF repeat and discoidin-domain protein involved in sperm-egg binding. Cell.

[190] Shur B.D., Ensslin M.A., Rodeheffer C. (2004). SED1 function during mammalian sperm-egg adhesion. Curr. Opin. Cell Biol..

[191] Copland S.D., Murphy A.A., Shur B.D. (2009). The mouse gamete adhesin, SED1, is expressed on the surface of acrosome-intact human sperm. Fertil. Steril..

[192] Ensslin M., Vogel T., Calvete J.J., Thole H.H., Schmidtke J., Matsuda T., Topfer-Petersen E. (1998). Molecular cloning and characterization of P47, a novel boar sperm-associated zona pellucida-binding protein homologous to a family of mammalian secretory proteins. Biol. Reprod..

[193] Petrunkina A.M., Lakamp A., Gentzel M., Ekhlasi-Hundrieser M., Topfer-Petersen E. (2003). Fate of lactadherin P47 during post-testicular maturation and capacitation of boar spermatozoa. Reproduction.

[194] Hagaman J.R., Moyer J.S., Bachman E.S., Sibony M., Magyar P.L., Welch J.E., Smithies O., Krege J.H., O’Brien D.A. (1998). Angiotensin-converting enzyme and male fertility. Proc. Natl. Acad. Sci. USA.

[195] Ramaraj P., Kessler S.P., Colmenares C., Sen G.C. (1998). Selective restoration of male fertility in mice lacking angiotensin-converting enzymes by sperm-specific expression of the testicular isozyme. J. Clin. Investig..

[196] Foresta C., Indino M., Manoni F., Scandellari C. (1987). Angiotensin-converting enzyme content of human spermatozoa and its release during capacitation. Fertil. Steril..

[197] Köhn F.M., Miska W., Schill W.B. (1995). Release of angiotensin-converting enzyme (ACE) from human spermatozoa during capacitation and acrosome reaction. J. Androl..

[198] Köhn F.M., Dammshäuser I., Neukamm C., Renneberg H., Siems W.E., Schill W.B., Aumüller G. (1998). Ultrastructural localization of angiotensin-converting enzyme in ejaculated human spermatozoa. Hum. Reprod..

[199] Pilch B., Mann M. (2006). Large-scale and high-confidence proteomic analysis of human seminal plasma. Genome Biol..

[200] Yotsumoto H., Sato S., Shibuya M. (1984). Localization of angiotensin converting enzyme (dipeptidyl carboxypeptidase) in swine sperm by immunofluorescence. Life Sci..

[201] Gatti J.L., Druart X., Guerin Y., Dacheux F., Dacheux J.L. (1999). A 105- to 94-kilodalton protein in the epididymal fluids of domestic mammals is angiotensin I-converting enzyme (ACE); evidence that sperm are the source of this ACE. Biol. Reprod..

[202] Druart X., Rickard J.P., Mactier S., Kohnke P.L., Kershaw-Young C.M., Bathgate R., Gibb Z., Crossett B., Tsikis G., Labas V. (2013). Proteomic characterization and cross species comparison of mammalian seminal plasma. J. Proteom..

[203] Zigo M., Jonakova V., Sulc M., Manaskova-Postlerova P. (2013). Characterization of sperm surface protein patterns of ejaculated and capacitated boar sperm, with the detection of ZP binding candidates. Int. J. Biol. Macromol..

[204] Costa D.S., Thundathil J.C. (2012). Characterization and activity of angiotensin-converting enzyme in Holstein semen. Anim. Reprod. Sci..

[205] Ojaghi M., Kastelic J., Thundathil J. (2017). Testis-specific isoform of angiotensin-converting enzyme (tACE) is involved in the regulation of bovine sperm capacitation. Mol. Reprod. Dev..

[206] Ojaghi M., Kastelic J., Thundathil J.C. (2018). Testis-specific isoform of angiotensin-converting enzyme (tACE) as a candidate marker for bull fertility. Reprod. Fertil. Dev..

[207] Bleil J.D., Wassarman P.M. (1990). Identification of a ZP3-binding protein on acrosome-intact mouse sperm by photoaffinity crosslinking. Proc. Natl. Acad. Sci. USA.

[208] Cheng A., Le T., Palacios M., Bookbinder L.H., Wassarman P.M., Suzuki F., Bleil J.D. (1994). Sperm-egg recognition in the mouse: Characterization of sp56, a sperm protein having specific affinity for ZP3. J. Cell Biol..

[209] Kim K.S., Cha M.C., Gerton G.L. (2001). Mouse sperm protein sp56 is a component of the acrosomal matrix. Biol. Reprod..

[210] Muro Y., Buffone M.G., Okabe M., Gerton G.L. (2012). Function of the acrosomal matrix: Zona pellucida 3 receptor (ZP3R/sp56) is not essential for mouse fertilization. Biol. Reprod..

[211] Sullivan R., Bleau G. (1985). Interaction between isolated components from mammalian sperm and egg. Gamete Res..

[212] Sullivan R., Robitaille G. (1989). Heterogeneity of epididymal spermatozoa of the hamster. Gamete Res..

[213] Bérubé B., Sullivan R. (1994). Inhibition of in vivo fertilization by active immunization of male hamsters against a 26-kDa sperm glycoprotein. Biol. Reprod..

[214] Bégin S., Bérubé B., Boué F., Sullivan R. (1995). Comparative immunoreactivity of mouse and hamster sperm proteins recognized by an anti-P26h hamster sperm protein. Mol. Reprod. Dev..

[215] Boué F., Bérubé B., De Lamirande E., Gagnon C., Sullivan R. (1994). Human sperm-zona pellucida interaction is inhibited by an antiserum against a hamster sperm protein. Biol. Reprod..

[216] Boué F., Blais J., Sullivan R. (1996). Surface localization of P34H an epididymal protein, during maturation, capacitation, and acrosome reaction of human spermatozoa. Biol. Reprod..

[217] Van Gestel R.A., Brewis I.A., Ashton P.R., Brouwers J.F., Gadella B.M. (2007). Multiple proteins present in purified porcine sperm apical plasma membranes interact with the zona pellucida of the oocyte. Mol. Hum. Reprod..

[218] Parent S., Lefievre L., Brindle Y., Sullivan R. (1998). Bull subfertility is associated with low levels of a sperm membrane antigen. Mol. Reprod. Dev..

[219] Lessard C., Parent S., Leclerc P., Bailey J.L., Sullivan R. (2000). Cryopreservation alters the levels of the bull sperm surface protein P25b. J. Androl..

[220] Frenette G., Sullivan R. (2001). Prostasome-like particles are involved in the transfer of P25b from the bovine epididymal fluid to the sperm surface. Mol. Reprod. Dev..

[221] Sanz L., Calvete J.J., Jonakova V., Topfer-Petersen E. (1992). Boar spermadhesins AQN-1 and AWN are sperm-associated acrosin inhibitor acceptor proteins. FEBS Lett..

[222] Sanz L., Calvete J.J., Schäfer W., Mann K., Töpfer-Petersen E. (1992). Isolation and biochemical characterization of two isoforms of a boar sperm zona pellucida-binding protein. Biochim. Biophys. Acta (BBA) Gene Struct. Expr..

[223] Veselsky L., Jonakova V., Sanz M.L., Topfer-Petersen E., Cechova D. (1992). Binding of a 15 kDa glycoprotein from spermatozoa of boars to surface of zona pellucida and cumulus oophorus cells. J. Reprod. Fertil..

[224] Dostalova Z., Calvete J.J., Topfer-Petersen E. (1995). Interaction of non-aggregated boar AWN-1 and AQN-3 with phospholipid matrices. A model for coating of spermadhesins to the sperm surface. Biol. Chem..

[225] Ensslin M., Calvete J.J., Thole H.H., Sierralta W.D., Adermann K., Sanz L., Topfer-Petersen E. (1995). Identification by affinity chromatography of boar sperm membrane-associated proteins bound to immobilized porcine zona pellucida. Mapping of the phosphorylethanolamine-binding region of spermadhesin AWN. Biol. Chem..

[226] Calvete J.J., Carrera E., Sanz L., Töpfer-Petersen E. (1996). Boar spermadhesins AQN-1 and AQN-3: Oligosaccharide and zona pellucida binding characteristics. Biol. Chem..

[227] Jonakova V., Kraus M., Veselsky L., Cechova D., Bezouska K., Ticha M. (1998). Spermadhesins of the AQN and AWN families, DQH sperm surface protein and HNK protein in the heparin-binding fraction of boar seminal plasma. J. Reprod. Fertil..

[228] Veselsky L., Peknicova J., Cechova D., Kraus M., Geussova G., Jonakova V. (1999). Characterization of boar spermadhesins by monoclonal and polyclonal antibodies and their role in binding to oocytes. Am. J. Reprod. Immunol..

[229] Petrunkina A.M., Harrison R.A., Topfer-Petersen E. (2000). Only low levels of spermadhesin AWN are detectable on the surface of live ejaculated boar spermatozoa. Reprod. Fertil. Dev..

[230] Tichá M., Kraus M., Cechová D., Jonáková V. (1998). Saccharide-binding properties of boar AQN spermadhesins and DQH sperm surface protein. Folia Biol..

[231] Jonakova V., Manaskova P., Kraus M., Liberda J., Ticha M. (2000). Sperm surface proteins in mammalian fertilization. Mol. Reprod. Dev..

[232] Manaskova P., Peknicova J., Elzeinova F., Ticha M., Jonakova V. (2007). Origin, localization and binding abilities of boar DQH sperm surface protein tested by specific monoclonal antibodies. J. Reprod. Immunol..

[233] Liberda J., Ryslavá H., Jelínková P., Jonáková V., Tichá M. (2002). Affinity chromatography of bull seminal proteins on mannan-Sepharose. J. Chromatogr. B.

[234] Lin Y.N., Roy A., Yan W., Burns K.H., Matzuk M.M. (2007). Loss of zona pellucida binding proteins in the acrosomal matrix disrupts acrosome biogenesis and sperm morphogenesis. Mol. Cell. Biol..

[235] Yu Y., Vanhorne J., Oko R. (2009). The origin and assembly of a zona pellucida binding protein, IAM38, during spermiogenesis. Microsc. Res. Tech..

[236] Yatsenko A.N., O’Neil D.S., Roy A., Arias-Mendoza P.A., Chen R., Murthy L.J., Lamb D.J., Matzuk M.M. (2012). Association of mutations in the zona pellucida binding protein 1 (ZPBP1) gene with abnormal sperm head morphology in infertile men. Mol. Hum. Reprod..

[237] Guo Y., Jiang J., Zhang H., Wen Y., Zhang H., Cui Y., Tian J., Jiang M., Liu X., Wang G. (2019). Proteomic Analysis of Dpy19l2-Deficient Human Globozoospermia Reveals Multiple Molecular Defects. Proteom. Clin. Appl..

[238] Mori E., Baba T., Iwamatsu A., Mori T. (1993). Purification and characterization of a 38-kDa protein, sp38, with zona pellucida-binding property from porcine epididymal sperm. Biochem. Biophys. Res. Commun..

[239] Mori E., Kashiwabara S., Baba T., Inagaki Y., Mori T. (1995). Amino acid sequences of porcine Sp38 and proacrosin required for binding to the zona pellucida. Dev. Biol..

[240] Yu Y., Xu W., Yi Y.J., Sutovsky P., Oko R. (2006). The extracellular protein coat of the inner acrosomal membrane is involved in zona pellucida binding and penetration during fertilization: Characterization of its most prominent polypeptide (IAM38). Dev. Biol..

[241] Dubova-Mihailova M., Mollova M., Ivanova M., Kehayov I., Kyurkchiev S. (1991). Identification and characterization of human acrosomal antigen defined by a monoclonal antibody with blocking effect on in vitro fertilization. J. Reprod. Immunol..

[242] Foster J.A., Klotz K.L., Flickinger C.J., Thomas T.S., Wright R.M., Castillo J.R., Herr J.C. (1994). Human SP-10: Acrosomal distribution, processing, and fate after the acrosome reaction. Biol. Reprod..

[243] Hamatani T., Tanabe K., Kamei K., Sakai N., Yamamoto Y., Yoshimura Y. (2000). A monoclonal antibody to human SP-10 inhibits in vitro the binding of human sperm to hamster oolemma but not to human Zona pellucida. Biol. Reprod..

[244] Coonrod S.A., Herr J.C., Westhusin M.E. (1996). Inhibition of bovine fertilization in vitro by antibodies to SP-10. J. Reprod. Fertil..

[245] Avilés M., Abascal I., Martínez-Menárguez J.A., Castells M.T., Skalaban S.R., Ballesta J., Alhadeff J.A. (1996). Immunocytochemical localization and biochemical characterization of a novel plasma membrane-associated, neutral pH optimum alpha-L-fucosidase from rat testis and epididymal spermatozoa. Biochem. J..

[246] Phopin K., Nimlamool W., Bartlett M.J., Bean B.S. (2012). Distribution, crypticity, stability, and localization of α-L-fucosidase of mouse cauda epididymal sperm. Mol. Reprod. Dev..

[247] Phopin K., Nimlamool W., Lowe-Krentz L.J., Douglass E.W., Taroni J.N., Bean B.S. (2013). Roles of mouse sperm-associated alpha-L-fucosidases in fertilization. Mol. Reprod. Dev..

[248] Venditti J.J., Donigan K.A., Bean B.S. (2007). Crypticity and functional distribution of the membrane associated alpha-L-fucosidase of human sperm. Mol. Reprod. Dev..

[249] Venditti J.J., Bean B.S. (2009). Stabilization of membrane-associated alpha-L-fucosidase by the human sperm equatorial segment. Int. J. Androl..

[250] Jauhiainen A., Vanha-Perttula T. (1986). alpha-L-Fucosidase in the reproductive organs and seminal plasma of the bull. Biochim. Biophys. Acta (BBA) Gene Struct. Expr..

[251] Peterson R.N., Hunt W.P. (1989). Identification, isolation, and properties of a plasma membrane protein involved in the adhesion of boar sperm to the porcine zona pellucida. Gamete Res..

[252] Zayas-Perez H., Casas E., Bonilla E., Betancourt M. (2005). Inhibition of sperm-zona pellucida binding by a 55 kDa pig sperm protein in vitro. Arch. Androl..

[253] Redgrove K.A., Anderson A.L., Dun M.D., McLaughlin E.A., O’Bryan M.K., Aitken R.J., Nixon B. (2011). Involvement of multimeric protein complexes in mediating the capacitation-dependent binding of human spermatozoa to homologous zonae pellucidae. Dev. Biol..

[254] Kongmanas K., Kruevaisayawan H., Saewu A., Sugeng C., Fernandes J., Souda P., Angel J.B., Faull K.F., Aitken R.J., Whitelegge J. (2015). Proteomic Characterization of Pig Sperm Anterior Head Plasma Membrane Reveals Roles of Acrosomal Proteins in ZP3 Binding. J. Cell. Physiol..

[255] Shur B.D., Bennett D. (1979). A specific defect in galactosyltransferase regulation on sperm bearing mutant alleles of the T/t locus. Dev. Biol..

[256] Lopez L.C., Bayna E.M., Litoff D., Shaper N.L., Shaper J.H., Shur B.D. (1985). Receptor function of mouse sperm surface galactosyltransferase during fertilization. J. Cell Biol..

[257] Nixon B., Lu Q., Wassler M.J., Foote C.I., Ensslin M.A., Shur B.D. (2001). Galactosyltransferase function during mammalian fertilization. Cells Tissues Organs.

[258] Fayrer-Hosken R.A., Caudle A.B., Shur B.D. (1991). Galactosyltransferase activity is restricted to the plasma membranes of equine and bovine sperm. Mol. Reprod. Dev..

[259] Lu Q., Shur B.D. (1997). Sperm from beta 1,4-galactosyltransferase-null mice are refractory to ZP3-induced acrosome reactions and penetrate the zona pellucida poorly. Development.

[260] Lyng R., Shur B.D. (2007). Sperm-egg binding requires a multiplicity of receptor-ligand interactions: New insights into the nature of gamete receptors derived from reproductive tract secretions. Soc. Reprod. Fertil. Suppl..

[261] Topfer-Petersen E., Friess A.E., Nguyen H., Schill W.B. (1985). Evidence for a fucose-binding protein in boar spermatozoa. Histochemistry.

[262] Topfer-Petersen E., Henschen A. (1987). Acrosin shows zona and fucose binding, novel properties for a serine proteinase. FEBS Lett..

[263] Baba T., Azuma S., Kashiwabara S., Toyoda Y. (1994). Sperm from mice carrying a targeted mutation of the acrosin gene can penetrate the oocyte zona pellucida and effect fertilization. J. Biol. Chem..

[264] Isotani A., Matsumura T., Ogawa M., Tanaka T., Yamagata K., Ikawa M., Okabe M. (2017). A delayed sperm penetration of cumulus layers by disruption of acrosin gene in rats. Biol. Reprod..

[265] Adham I.M., Nayernia K., Engel W. (1997). Spermatozoa lacking acrosin protein show delayed fertilization. Mol. Reprod. Dev..

[266] Dudkiewicz A.B. (1983). Inhibition of fertilization in the rabbit by anti-acrosin antibodies. Mol. Reprod. Dev..

[267] Liu D.Y., Baker H.W. (1993). Inhibition of acrosin activity with a trypsin inhibitor blocks human sperm penetration of the zona pellucida. Biol. Reprod..

[268] Hirose M., Honda A., Fulka H., Tamura-Nakano M., Matoba S., Tomishima T., Mochida K., Hasegawa A., Nagashima K., Inoue K. (2020). Acrosin is essential for sperm penetration through the zona pellucida in hamsters. Proc. Natl. Acad. Sci. USA.

[269] Yamagata K., Honda A., Kashiwabara S.I., Baba T. (1999). Difference of acrosomal serine protease system between mouse and other rodent sperm. Dev. Genet..

[270] Wassarman P.M. (2008). Zona pellucida glycoproteins. J. Biol. Chem..

[271] Marco-Jiménez F., Naturil-Alfonso C., Jiménez-Trigos E., Lavara R., Vicente J.S. (2012). Influence of zona pellucida thickness on fertilization, embryo implantation and birth. Anim. Reprod. Sci..

[272] Lamas-Toranzo I., Fonseca Balvís N., Querejeta-Fernández A., Izquierdo-Rico M.J., González-Brusi L., Lorenzo P.L., García-Rebollar P., Avilés M., Bermejo-Álvarez P. (2019). ZP4 confers structural properties to the zona pellucida essential for embryo development. Elife.

[273] Abe H., Oikawa T. (1990). Ultrastructural evidence for an association between an oviductal glycoprotein and the zona pellucida of the golden hamster egg. J. Exp. Zool..

[274] Wiesak T., Wasielak M., Złotkowska A., Milewski R. (2017). Effect of vitrification on the zona pellucida hardening and follistatin and cathepsin B genes expression and developmental competence of in vitro matured bovine oocytes. Cryobiology.

[275] Balakier H., Sojecki A., Motamedi G., Bashar S., Mandel R., Librach C. (2012). Is the zona pellucida thickness of human embryos influenced by women’s age and hormonal levels?. Fertil. Steril..

[276] Herlyn H., Zischler H. (2008). The molecular evolution of sperm zonadhesin. Int. J. Dev. Biol..

[277] Tardif S., Cormier N. (2011). Role of zonadhesin during sperm-egg interaction: A species-specific acrosomal molecule with multiple functions. Mol. Hum. Reprod..

[278] Hardy D.M., Garbers D.L. (1994). Species-specific binding of sperm proteins to the extracellular matrix (zona pellucida) of the egg. J. Biol. Chem..

[279] Hickox J.R., Bi M., Hardy D.M. (2001). Heterogeneous processing and zona pellucida binding activity of pig zonadhesin. J. Biol. Chem..

[280] Dudkiewicz A.B. (1984). Purification of boar acrosomal arylsulfatase A and possible role in the penetration of cumulus cells. Biol. Reprod..

[281] White D., Weerchatyanukul W., Gadella B.M., Kamolvarin N., Attar M., Tanphaichitr N. (2000). Role of sperm sulfogalactosylglycerolipid in mouse sperm-zona pellucida binding. Biol. Reprod..

[282] Rattanachaiyanont M., Weerachatyanukul W., Leveille M.-C., Taylor T., D’Amours D., Rivers D., Leader A., Tanphaichitr N. (2001). Anti-SLIP1-reactive proteins exist on human sperm and are involved in zona-pellucida binding. Mol. Hum. Reprod..

[283] Weerachatyanukul W., Xu H., Anupriwan A., Carmona E., Wade M., Hermo L., da Silva S.M., Rippstein P., Sobhon P., Sretarugsa P. (2003). Acquisition of arylsulfatase A onto the mouse sperm surface during epididymal transit. Biol. Reprod..

[284] Schenk M., Koppisetty C.A., Santos D.C., Carmona E., Bhatia S., Nyholm P.G., Tanphaichitr N. (2009). Interaction of arylsulfatase-A (ASA) with its natural sulfoglycolipid substrates: A computational and site-directed mutagenesis study. Glycoconj. J..

[285] Gadella B.M., Colenbrander B., Golde L.M.v., Lopes-Cardozo M. (1993). Boar seminal vesicles secrete arylsulfatases into seminal plasma: Evidence that desulfation of seminolipid occurs only after ejaculation. Biol. Reprod..

[286] Carmona E., Weerachatyanukul W., Xu H., Fluharty A., Anupriwan A., Shoushtarian A., Chakrabandhu K., Tanphaichitr N. (2002). Binding of arylsulfatase A to mouse sperm inhibits gamete interaction and induces the acrosome reaction. Biol. Reprod..

[287] Gonzalez-Cadavid V., Martins J.A., Moreno F.B., Andrade T.S., Santos A.C., Monteiro-Moreira A.C., Moreira R.A., Moura A.A. (2014). Seminal plasma proteins of adult boars and correlations with sperm parameters. Theriogenology.

[288] Silva E., Frost D., Li L., Bovin N., Miller D.J. (2017). Lactadherin is a candidate oviduct Lewis X trisaccharide receptor on porcine spermatozoa. Andrology.

[289] Zigo M., Manaskova-Postlerova P., Jonakova V., Kerns K., Sutovsky P. (2019). Compartmentalization of the proteasome-interacting proteins during sperm capacitation. Sci. Rep..

[290] Miles E.L., O’Gorman C., Zhao J., Samuel M., Walters E., Yi Y.J., Sutovsky M., Prather R.S., Wells K.D., Sutovsky P. (2013). Transgenic pig carrying green fluorescent proteasomes. Proc. Natl. Acad. Sci. USA.

[291] Zimmerman S.W., Manandhar G., Yi Y.J., Gupta S.K., Sutovsky M., Odhiambo J.F., Powell M.D., Miller D.J., Sutovsky P. (2011). Sperm proteasomes degrade sperm receptor on the egg zona pellucida during mammalian fertilization. PLoS ONE.

[292] Zigo M., Jonakova V., Manaskova-Postlerova P., Kerns K., Sutovsky P. (2019). Ubiquitin-proteasome system participates in the de-aggregation of spermadhesin and DQH protein during boar sperm capacitation. Reproduction.

[293] Wassarman P.M., Jovine L., Litscher E.S. (2001). A profile of fertilization in mammals. Nat. Cell Biol..

[294] Foster J.A., Friday B.B., Maulit M.T., Blobel C., Winfrey V.P., Olson G.E., Kim K.S., Gerton G.L. (1997). AM67, a secretory component of the guinea pig sperm acrosomal matrix, is related to mouse sperm protein sp56 and the complement component 4-binding proteins. J. Biol. Chem..

[295] Kim K.S., Foster J.A., Gerton G.L. (2001). Differential release of guinea pig sperm acrosomal components during exocytosis. Biol. Reprod..

[296] Buffone M.G., Zhuang T., Ord T.S., Hui L., Moss S.B., Gerton G.L. (2008). Recombinant mouse sperm ZP3-binding protein (ZP3R/sp56) forms a high order oligomer that binds eggs and inhibits mouse fertilization in vitro. J. Biol. Chem..

[297] Da Ros V.G., Maldera J.A., Willis W.D., Cohen D.J., Goulding E.H., Gelman D.M., Rubinstein M., Eddy E.M., Cuasnicu P.S. (2008). Impaired sperm fertilizing ability in mice lacking Cysteine-RIch Secretory Protein 1 (CRISP1). Dev. Biol..

[298] Naz R.K., Ahmad K. (1994). Molecular identities of human sperm proteins that bind human zona pellucida: Nature of sperm-zona interaction, tyrosine kinase activity, and involvement of FA-1. Mol. Reprod. Dev..

[299] Naz R.K., Bhargava K.K. (1990). Antibodies to sperm surface fertilization antigen (FA-1): Their specificities and site of interaction with sperm in male genital tract. Mol. Reprod. Dev..

[300] Pan P.P., Zhan Q.T., Le F., Zheng Y.M., Jin F. (2013). Angiotensin-converting enzymes play a dominant role in fertility. Int. J. Mol. Sci..

[301] Castilho C.S., Fontes P.K., Franchi F.F., Santos P.H., Razza E.M., Tolekova A. (2017). Renin-Angiotensin System on Reproductive Biology. Renin-Angiotensin System Past, Present and Future.

[302] Reis A.B., Araújo F.C., Pereira V.M., Dos Reis A.M., Santos R.A., Reis F.M. (2010). Angiotensin (1–7) and its receptor Mas are expressed in the human testis: Implications for male infertility. J. Mol. Histol..

[303] Wang Z., Xu X. (2020). scRNA-seq Profiling of Human Testes Reveals the Presence of the ACE2 Receptor, A Target for SARS-CoV-2 Infection in Spermatogonia, Leydig and Sertoli Cells. Cells.

[304] Shang J., Ye G., Shi K., Wan Y., Luo C., Aihara H., Geng Q., Auerbach A., Li F. (2020). Structural basis of receptor recognition by SARS-CoV-2. Nature.

[305] Sullivan R., Saez F., Girouard J., Frenette G. (2005). Role of exosomes in sperm maturation during the transit along the male reproductive tract. Blood Cells Mol. Dis..

[306] Petit F.M., Serres C., Bourgeon F., Pineau C., Auer J. (2013). Identification of sperm head proteins involved in zona pellucida binding. Hum. Reprod..

[307] Feiden S., Wolfrum U., Wegener G., Kamp G. (2008). Expression and compartmentalisation of the glycolytic enzymes GAPDH and pyruvate kinase in boar spermatogenesis. Reprod. Fertil. Dev..

[308] Topfer-Petersen E., Romero A., Varela P.F., Ekhlasi-Hundrieser M., Dostalova Z., Sanz L., Calvete J.J. (1998). Spermadhesins: A new protein family. Facts, hypotheses and perspectives. Andrologia.

[309] Jonakova V., Ticha M. (2004). Boar seminal plasma proteins and their binding properties. Collect. Czechoslov. Chem. Commun..

[310] Jonakova V., Manaskova P., Ticha M. (2007). Separation, characterization and identification of boar seminal plasma proteins. J. Chromatogr. B.

[311] Jonakova V., Jonak J., Ticha M., Jiang Z., Ott T.L. (2010). Proteomics of Male Seminal Plasma. Reproductive Genomics in Domestic Animals.

[312] Ekhlasi-Hundrieser M., Gohr K., Wagner A., Tsolova M., Petrunkina A., Topfer-Petersen E. (2005). Spermadhesin AQN1 is a candidate receptor molecule involved in the formation of the oviductal sperm reservoir in the pig. Biol. Reprod..

[313] Calvete J.J., Raida M., Gentzel M., Urbanke C., Sanz L., Topfer-Petersen E. (1997). Isolation and characterization of heparin- and phosphorylcholine-binding proteins of boar and stallion seminal plasma. Primary structure of porcine pB1. FEBS Lett..

[314] Bezouska K., Sklenár J., Novák P., Halada P., Havlícek V., Kraus M., Tichá M., Jonáková V. (1999). Determination of the complete covalent structure of the major glycoform of DQH sperm surface protein, a novel trypsin-resistant boar seminal plasma O-glycoprotein related to pB1 protein. Protein Sci..

[315] Fan J., Lefebvre J., Manjunath P. (2006). Bovine seminal plasma proteins and their relatives: A new expanding superfamily in mammals. Gene.

[316] Plante G., Prud’homme B., Fan J., Lafleur M., Manjunath P. (2016). Evolution and function of mammalian binder of sperm proteins. Cell Tissue Res..

[317] Kim E., Park K.E., Kim J.S., Baek D.C., Lee J.W., Lee S.R., Kim M.S., Kim S.H., Kim C.S., Koo D.B. (2009). Importance of the porcine ADAM3 disintegrin domain in sperm-egg interaction. J. Reprod. Dev..

[318] Mori E., Fukuda H., Imajoh-Ohmi S., Mori T., Takasaki S. (2012). Purification of N-acetyllactosamine-binding activity from the porcine sperm membrane: Possible involvement of an ADAM complex in the carbohydrate-binding activity of sperm. J. Reprod. Dev..

[319] Srivastava N., Jerome A., Srivastava S.K., Ghosh S.K., Kumar A. (2013). Bovine seminal PDC-109 protein: An overview of biochemical and functional properties. Anim. Reprod. Sci..

[320] Gwathmey T.M., Ignotz G.G., Suarez S.S. (2003). PDC-109 (BSP-A1/A2) promotes bull sperm binding to oviductal epithelium in vitro and may be involved in forming the oviductal sperm reservoir. Biol. Reprod..

[321] Somashekar L., Selvaraju S., Parthipan S., Ravindra J.P. (2015). Profiling of sperm proteins and association of sperm PDC-109 with bull fertility. Syst. Biol. Reprod. Med..

[322] Kumar P., Kumar D., Singh I., Yadav P.S. (2012). Seminal Plasma Proteome: Promising Biomarkers for Bull Fertility. Agric. Res..

[323] Kelly V.C., Kuy S., Palmer D.J., Xu Z., Davis S.R., Cooper G.J. (2006). Characterization of bovine seminal plasma by proteomics. Proteomics.

[324] Redgrove K.A., Aitken R.J., Nixon B., Abdelmohsen K. (2012). More Than a Simple Lock and Key Mechanism: Unraveling the Intricacies of Sperm-Zona Pellucida Binding. Binding Protein.

[325] van Gestel R.A., Brewis I.A., Ashton P.R., Helms J.B., Brouwers J.F., Gadella B.M. (2005). Capacitation-dependent concentration of lipid rafts in the apical ridge head area of porcine sperm cells. Mol. Hum. Reprod..

[326] Bou Khalil M., Chakrabandhu K., Xu H., Weerachatyanukul W., Buhr M., Berger T., Carmona E., Vuong N., Kumarathasan P., Wong P.T. (2006). Sperm capacitation induces an increase in lipid rafts having zona pellucida binding ability and containing sulfogalactosylglycerolipid. Dev. Biol..

[327] Gadella B.M., Tsai P.S., Boerke A., Brewis I.A. (2008). Sperm head membrane reorganisation during capacitation. Int. J. Dev. Biol..

[328] Simons K., Sampaio J.L. (2011). Membrane organization and lipid rafts. Cold Spring Harb. Perspect. Biol..

[329] Pike L.J. (2006). Rafts defined: A report on the Keystone Symposium on Lipid Rafts and Cell Function. J. Lipid Res..

[330] Tanphaichitr N., Bou Khalil M., Weerachatyanukul W., Kates M., Xu H., Carmona E., Attar M., Carrier D., De Vriese S.R., Christophe A.B. (2003). Physiological and Biophysical Properties of Male Germ Cell Sulfogalactosylglycerolipid. Male Fertility and Lipid Metabolsim.

[331] Tanphaichitr N., Kongmanas K., Faull K.F., Whitelegge J., Compostella F., Goto-Inoue N., Linton J.J., Doyle B., Oko R., Xu H. (2018). Properties, metabolism and roles of sulfogalactosylglycerolipid in male reproduction. Prog. Lipid Res..

[332] Attar M., Kates M., Bou Khalil M., Carrier D., Wong P.T., Tanphaichitr N. (2000). A Fourier-transform infrared study of the interaction between germ-cell specific sulfogalactosylglycerolipid and dimyristoylglycerophosphocholine. Chem. Phys. Lipids.

[333] Weerachatyanukul W., Probodh I., Kongmanas K., Tanphaichitr N., Johnston L.J. (2007). Visualizing the localization of sulfoglycolipids in lipid raft domains in model membranes and sperm membrane extracts. Biochim. Biophys. Acta (BBA) Gene Struct. Expr..

[334] Weerachatyanukul W., Rattanachaiyanont M., Carmona E., Furimsky A., Mai A., Shoushtarian A., Sirichotiyakul S., Ballakier H., Leader A., Tanphaichitr N. (2001). Sulfogalactosylglycerolipid is involved in human gamete interaction. Mol. Reprod. Dev..

[335] Hartl F.U., Bracher A., Hayer-Hartl M. (2011). Molecular chaperones in protein folding and proteostasis. Nature.

[336] Kim Y.E., Hipp M.S., Bracher A., Hayer-Hartl M., Hartl F.U. (2013). Molecular chaperone functions in protein folding and proteostasis. Annu. Rev. Biochem..

[337] Asquith K.L., Baleato R.M., McLaughlin E.A., Nixon B., Aitken R.J. (2004). Tyrosine phosphorylation activates surface chaperones facilitating sperm-zona recognition. J. Cell Sci..

[338] Kamaruddin M., Kroetsch T., Basrur P.K., Hansen P.J., King W.A. (2004). Immunolocalization of heat shock protein 70 in bovine spermatozoa. Andrologia.

[339] Spinaci M., Volpe S., Bernardini C., De Ambrogi M., Tamanini C., Seren E., Galeati G. (2005). Immunolocalization of heat shock protein 70 (Hsp 70) in boar spermatozoa and its role during fertilization. Mol. Reprod. Dev..

[340] Nixon B., Aitken R.J. (2009). The biological significance of detergent-resistant membranes in spermatozoa. J. Reprod. Immunol..

[341] Nixon B., Bielanowicz A., McLaughlin E.A., Tanphaichitr N., Ensslin M.A., Aitken R.J. (2009). Composition and significance of detergent resistant membranes in mouse spermatozoa. J. Cell. Physiol..

[342] Naaby-Hansen S., Herr J.C. (2010). Heat shock proteins on the human sperm surface. J. Reprod. Immunol..

[343] Asquith K.L., Harman A.J., McLaughlin E.A., Nixon B., Aitken R.J. (2005). Localization and significance of molecular chaperones, heat shock protein 1, and tumor rejection antigen gp96 in the male reproductive tract and during capacitation and acrosome reaction. Biol. Reprod..

[344] Walsh A., Whelan D., Bielanowicz A., Skinner B., Aitken R.J., O’Bryan M.K., Nixon B. (2008). Identification of the molecular chaperone, heat shock protein 1 (chaperonin 10), in the reproductive tract and in capacitating spermatozoa in the male mouse. Biol. Reprod..

[345] Dun M.D., Smith N.D., Baker M.A., Lin M., Aitken R.J., Nixon B. (2011). The chaperonin containing TCP1 complex (CCT/TRiC) is involved in mediating sperm-oocyte interaction. J. Biol. Chem..

[346] Bernabò N., Palestini P., Botto L., Mattioli M., Barboni B., Erickson B.T. (2014). Membrane Dynamics Occuring during Capacitation of Mammalian Spermatozoa. Spermatozoa. Biology, Motility and Function and Chromosomal Abnormalities.

[347] Sutovsky P. (2011). Sperm proteasome and fertilization. Reproduction.

[348] Kerns K., Morales P., Sutovsky P. (2016). Regulation of Sperm Capacitation by the 26S Proteasome: An Emerging New Paradigm in Spermatology. Biol. Reprod..

[349] Sasanami T., Sugiura K., Tokumoto T., Yoshizaki N., Dohra H., Nishio S., Mizushima S., Hiyama G., Matsuda T. (2012). Sperm proteasome degrades egg envelope glycoprotein ZP1 during fertilization of Japanese quail (*Coturnix japonica*). Reproduction.

[350] Sawada H., Mino M., Akasaka M. (2014). Sperm proteases and extracellular ubiquitin-proteasome system involved in fertilization of ascidians and sea urchins. Adv. Exp. Med. Biol..

